# Antiviral and immunomodulatory interferon-beta in high-risk COVID-19 patients: a structured summary of a study protocol for a randomised controlled trial

**DOI:** 10.1186/s13063-021-05367-6

**Published:** 2021-09-03

**Authors:** Eleonora Aricò, Luciano Castiello, Laura Bracci, Francesca Urbani, Flavia Lombardo, Ilaria Bacigalupo, Antonio Ancidoni, Nicola Vanacore, Alessandro Falcione, Chiara Reggiani, Giovanni Marco Dutti, Maria Grazia Maglie, Ombretta Papa, Pier Luigi Bartoletti, Giuseppina Ozzella, Nazario Bevilacqua, Emanuele Nicastri, Filippo Belardelli, Giuseppe Sconocchia

**Affiliations:** 1 FaBioCell, Core Facilities, Istituto Superiore di Sanità, viale Regina Elena 299, 00161 Rome, Italy; 2 Department of Oncology and Molecular Medicine, Istituto Superiore di Sanità, viale Regina Elena 299, 00161 Rome, Italy; 3 Medical Biotechnology and Translational Medicine PhD School, II University of Rome “Tor Vergata”, Via Montpellier 1, 00133 Rome, Italy; 4 National Centre for Disease Prevention and Health Promotion, Istituto Superiore di Sanità, viale Regina Elena 299, 00161 Rome, Italy; 5 Special Units for Regional Continued Care (USCAR), Rome, Italy; 6 Institute of Translational Pharmacology, National Research Council, Via Fosso del Cavaliere 100, 00133 Rome, Italy; 7 National Institute for Infectious Diseases “Lazzaro Spallanzani”, Via Portuense 292, 00149 Rome, Italy

**Keywords:** COVID-19, Randomised controlled trial, protocol, Interferon-beta, antiviral, immunomodulation, non-hospitalized patients

## Abstract

**Objectives:**

The primary objective of the study is to demonstrate the efficacy of low-dose IFN-β in reducing the risk of SARS-CoV-2 recently infected elderly patients to progress towards severe COVID-19 *versus* control group within 28 days. Secondary objectives are:

1. To assess the reduction in Intensive Care Unit (ICU) admission in patients treated with IFN-β *versus* control group within 28 days of randomization
2. To assess the reduction in number of deaths in IFN- β compared to control group (day 28)
3. To evaluate the increase in proportion of participants returning to negative SARS-CoV-2 RT-PCR in IFN-β -treated *versus* control group at Day 14 and Day 28
4. To assess the increase in SARS-CoV-2-specific binding antibody titers in IFN-β compared to control group (day 28)
5. To assess the safety of IFN-β -treated patients *versus* control group

**Trial design:**

Randomized, Open-Label, Controlled, Superiority Phase II Study. Patients, who satisfy all inclusion criteria and no exclusion criteria, will be randomly assigned to one of the two treatment groups in a ratio 2:1 (IFN-treated *versus* control patients). Randomization will be stratified by gender. Stratified randomization will balance the presence of male and female in both study arms.

**Participants:**

Male and female adults aged 65 years or older with newly diagnosed SARS-CoV-2 infection and mild COVID-19 symptoms are eligible for the study. The trial is being conducted in Rome.

Participants will be either hospitalized or home isolated. A group of physicians belonging to the Special Unit for Regional Continued Care (USCAR), specifically trained for the study and under the supervision of the National Institute for Infectious Diseases “Lazzaro Spallanzani”, will be responsible for the screening, enrolment, treatment and clinical monitoring of patients, thus acting as a bridge between clinical centers and territorial health management.

Inclusion criteria are as follows:

- ≥ 65 years of age at time of enrolment;
- Laboratory-confirmed SARS-CoV-2 infection as determined by PCR, in any specimen < 72 hours prior to randomization;
- Subject (or legally authorized representative) provides written informed consent prior to initiation of any study procedures;
- Understands and agrees to comply with planned study procedures;
- Agrees to the collection of nasopharyngeal swabs and venous blood samples per protocol;
- Being symptomatic for less than 7 days before starting therapy;
- NEWS2 score ≤2.

Exclusion criteria are as follows:

- Hospitalized patients with illness of any duration, and at least one of the following:
  - Clinical assessment (evidence of rales/crackles on exam) and SpO2 ≤ 94% on room air at rest or after walking test,
    OR
  - Acute respiratory failure requiring mechanical ventilation and/or supplemental oxygen;
- Patients currently using IFN-β (e.g., multiple sclerosis patients);
- Patients undergoing chemotherapy or other immunosuppressive treatments;
- Patients with chronic kidney diseases;
- Known allergy or hypersensitivity to IFN (including asthma);
- Any autoimmune disease (resulting from patient anamnesis);
- Patients with signs of dementia or neurocognitive disorders;
- Patients with current severe depression and/or suicidal ideations;
- Being concurrently involved in another clinical trial;
- HIV infection (based on the anamnesis);
- Use of any antiretroviral medication;
- Impaired renal function (eGFR calculated by CKD-EPI Creatinine equation < 30 ml/min);
- Presence of other severe diseases impairing life expectancy (e.g. patients are not expected to survive 28 days given their pre-existing medical condition);
- Any physical or psychological impediment in a patient that could let the investigator to suspect his/her poor compliance;
- Lack or withdrawal of informed consent

**Intervention and comparator:**

Control arm: No specific antiviral treatment besides standard of care.

Treatment arm: 11μg (3MIU) of IFN-β1a will be injected subcutaneously at day 1, 3, 7, and 10 in addition to standard of care. The drug solution, contained in a pre-filled cartridge, will be injected by means of the RebiSmart® electronic injection device.

Interferon β1a (Rebif®, Merck KGaA, Darmstadt, Germany) is a disease-modifying drug used to treat relapsing forms of multiple sclerosis (MS). The dose selected for this study is expected to exploit the antiviral and immunomodulatory properties of the cytokine without causing relevant toxicity or inducing refractoriness phenomena sometimes observed after high-dose and/or chronic IFNβ treatments.

**Main outcomes:**

Primary endpoint of the study is the proportion of patients experiencing a disease progression, during at least 5 days, according to the National Early Warning Score (NEWS2). The NEWS2 score is a standardized approach aimed at promptly detecting signs of clinical deterioration in acutely ill patients and establishing the potential need for higher level of care. It is based on the evaluation of vital signs, including respiratory rate, oxygen saturation, temperature, blood pressure, pulse/heart rate, AVPU response. The resulting observations, compared to a normal range, are combined in a single composite “alarm” score. Any other clinical sign clearly indicating a disease worsening will be considered as disease progression.

**Randomization:**

Sixty patients will be randomized 2:1 to receive IFN-β1a plus the standard of care or the standard of care only. Eligible patients will be randomized (no later than 36 h after enrolment) by means of a computerized central randomization system. All patients will receive a unique patient identification number at enrolling visit when signing the informed consent and before any study procedure is performed. This number remains constant throughout the entire study. The randomization of patients will be closed when 60 patients have been enrolled. The randomization will be stratified by sex; for each stratum a sequence of treatments randomly permuted in blocks of variable length (3 or 6) will be generated.

**Blinding (masking):**

This is an open-label study. After the randomization, patients will be notified whether they will be in the experimental arm or in the control arm.

**Numbers to be randomised (sample size):**

The study plans to enrol 60 patients: 40 in the IFN-β1a arm, 20 in the control arm, according to a 2:1 - treated: untreated ratio.

**Trial Status:**

Protocol Version: 3.0

Version Date: 18/03/2021

The study is open for recruitment since 16/04/2021.Recruitment is expected to l be completed before 15/08/2021.

**Trial registration:**

EudraCT N°: 2020-003872-42, registration date: 19/10/2020.

**Full protocol:**

The full protocol is attached as an additional file, accessible from the Trials website (Additional file 1). In the interest in expediting dissemination of this material, the familiar formatting has been eliminated; this Letter serves as a summary of the key elements of the full protocol.”

**Supplementary Information:**

The online version contains supplementary material available at 10.1186/s13063-021-05367-6.

## Supplementary Information

**Additional file 1.** Full study protocol.

## Data Availability

Not applicable

