# Supplementary material for: Antiviral and immunomodulatory interferon-beta in high-risk COVID-19 patients: a structured summary of a study protocol for a randomised controlled trial
Source: Trials. 2021 Sep 3;22:584. doi: 10.1186/s13063-021-05367-6 (PMC8413691; doi:10.1186/s13063-021-05367-6)
Supplement: Supplementary file 1 — Additional file 1. Full study protocol. [file 13063_2021_5367_MOESM1_ESM.pdf]

CLINICAL STUDY PROTOCOL

Study Title: Antiviral and Immunomodulatory Interferon-Beta in high-risk COVID-19 patients

Short title: (**ANT**iviral and **I**mmunomodulatory **I**nterferon-Beta in high-risk **C**ov**I**d-19 **PAT**i**E**nts) **ANTIICIPATE**

EudraCT N°: 2020-003872-42

Sponsor: Institute of Translational Pharmacology (IFT), National Research Council (CNR)

Sponsor Scientific Coordinator:

Filippo Belardelli,

IFT, CNR

Via Fosso del Cavaliere 100 - 00133 Rome - Italy

Phone: +39 06 4993 4486

Fax: +39 06 45488257

e-mail: filippo.belardelli@ift.cnr.it

Principal Investigator:

Emanuele Nicastri, MD

Istituto Nazionale per le Malattie Infettive Lazzaro Spallanzani, Via Portuense, 292 - 00149 Rome

Phone: +390655170393, Fax +390655170407

e-mail: emanuele.nicastri@inmi.it

Investigational Product Interferon β1a (Rebif™)

Clinical Study Phase: II

Version: 3.0

Issue Date: 18/03/2021

Protocol Signature form

Protocol Title:

Antiviral and Immunomodulatory Interferon-Beta in high-risk COVID-19 patients (ANTIICIPATE)

Version: 3.0

Version Date: 18/03/2021

I have read the protocol described below and agree to conduct this study in accordance with procedures described therein. I also agree to conduct the study in compliance with all applicable regulations.

____

Sponsor coordinator’s printed name

Giuseppe Sconocchia, MD

____

Principal Investigator’s printed name

Emanuele Nicastri, MD

Date: 18/03/2021

Summary

Protocol Signature form2

Summary4

List of abbreviations9

Roles and responsibilities:10

1. Synopsis14

    1.1 BACKGROUND14

    1.2 Objectives15

    1.3 Methodology16

    1.4 Expected results16

2. Background16

3. Rationale18

4. Impact for the National Health System18

5. Objectives of the study19

    5.1 Primary Objective20

        5.1.1 Primary endpoint and outcome20

    5.2 Secondary Objectives and Endpoints20

    5.3 Exploratory Endpoints21

        5.3.1 IFN-I Signaling21

        5.3.2 Cellular Immune-Monitoring22

        5.3.3 Systemic inflammation22

    5.4 Statistical hypothesis23

6. Study design24

7. Study Population24

    7.1 Case definition25

    7.2. Criteria for eligibility25

        7.2.1 Inclusion criteria25

        7.2.2 Exclusion criteria25

    7.3 Recruitment strategy26

8. Intervention27

    8.1 Experimental Drug and justification for dose27

    8.2 Treatment arms27

8.3 Standard patients monitoring28
8.4 Other therapies allowed29
8.5 Safety monitoring and individual stopping rules29
9. Methods30
9.1 Randomization30
9.2 Blinding30
9.3 Electronic case report form31
9.4. Safety Criteria Evaluation31
9.4.1 Safety profile31
9.4.2 Adverse events (AE) and serious adverse events (SAE)32
9.4.3 Regulatory reporting requirements for adverse events33
9.5 Secondary and Exploratory endpoints34
9.5.1 SARS-CoV-2 Antibodies34
9.5.2 Molecular IFN-I signaling34
9.5.3 Cellular Immune monitoring35
9.5.4 Systemic Inflammatory markers35
10. Statistical Plan35
11. Timing37
12. Feasibility37
13. Good clinical practices and ethics38
13.1. Good clinical practice38
13.2 Ethical aspects38
13.2.1 Written informed consent39
13.2.2 Subject data protection39
13.2.3 Audits and inspections40
13.2.4 Monitoring40
13.2.5 Declaration of interest40
13.2.6 Dissemination policy40
13.3 Insurance41
14. Budget42
15. Institutions agreement42
16. Participating Centers43
17. Publications and data properties43

18. References43

List of Appendices48

APPENDIX 1: Flow Chart of the Study49

APPENDIX 2: Timeline scheme50

APPENDIX 3: GANTT chart51

APPENDIX 4: eCRF design52

APPENDIX 5: Patient Diary and clinical record template61

APPENDIX 6: Standard operating procedure for drug management64

List of abbreviations

AE	Adverse event
AIFA	Italian medicines agency
ALT	Alanine AminoTransferase
ANCOVA	Analysis of CoVariance
ANOVA	Analysis of Variance
AVPU	Alert, Verbal, Pain, Unresponsive Score
AST	Aspartate AminoTransferase
CT	Coordination Team
CTCAE	Common Terminology Criteria for Adverse Events
CIM	Cellular Immune Monitoring
CKD-EPI	Chronic Kidney Disease Epidemiology Collaboration
CNR	Consiglio Nazionale delle Ricerche
COVID-19	Corona Virus 19 Disease
CRO	Contract Research Organization
CRP	C-Reactive Protein
EC	Ethical Committee
eCRF	electronic Case Report Form
eGFR	estimated Glomerular Filtration Rate
ELISA	Enzyme Linked ImmunoSorbent Assay
FFP	Filtering Face Mask
FKN	Fractalkine
GCP	Good Clinical Practice
Hb	Haemoglobin
ICAM-1	Intercellular Adhesion Molecule 1
ICH	International Conference on Harmonization
ICU	Intensive Care Unit
IEC	Independent Ethic Committee
IFT	CNR Institute of Translational Pharmacology
IFN	Interferon

IL-6	Interleukin-6
ITT	Intention To Treat
LDH	Lactate DeHydrogenase
LSRCHs	long-stay residential care homes
INMI	Istituto Nazionale Malattie Infettive
ISG	Interferon Stimulated Genes
ISS	Istituto Superiore di Sanità
IU	International Units
MAR	Missing At Random
MERS	Middle East respiratory syndrome
MFC	Multiparametric Flow Cytometry
MS	Multiple Sclerosis
NCI	National Cancer Institute
NEWS2	National Early Warning Score 2 (2017)
NK	Natural Killer
PBMC	Peripheral Blood Mononuclear Cell
PI	Principal Investigator
PP	Per Protocol
RCP	Riassunto delle Caratteristiche del Prodotto
RT-PCR	Real Time - Polymerase Chain Reaction
SAE	Serious Adverse Event
SARS	Severe Acute Respiratory Syndrome
SARS-CoV	SARS Corona Virus
SARS-Cov 2	New Corona Virus
SC	Steering Committee
SIM	Systemic Inflammatory Markers
SOCS	Suppressor of cytokine signaling
SpO_2	Oxygen Saturation
SUSAR	Suspected Unexpected Serious Adverse Reactions
TNF	Tumor Necrosis Factor

USCAR	Special Unit for regional continued care
VCAM-1	Vascular Cell Adhesion Molecule 1
VPA-1	Vascular Adhesion Protein 1
UBP43	Ubiquitin Protease 43
WBC	White Blood Cells
WHO	World Health Organization

Roles and responsibilities:

Principal investigator

Emanuele Nicastri, MD

National Institute for Infectious Diseases “Lazzaro Spallanzani”
Via Portuense 292, 00149 Rome, Italy
Phone: +390655170393, Fax +390655170407
e-mail: emanuele.nicastri@inmi.it

Co-Principal investigator

Pier Luigi Bartoletti, MD

Coordinator of the Special Units for Regional Continued Care (USCAR),
Phone: +390690253000,
e-mail: pl.bartoletti@gmail.com

Sponsor Coordinator

Giuseppe Sconocchia, MD

Institute of Translational Pharmacology (IFT)
National Research Council (CNR), Roma, Italy
Via Fosso del Cavaliere 100 - 00133 Rome - Italy
Phone: +390649934487; +390649934486
e-mail: giuseppe.sconocchia@ift.cnr.it
Responsible for the coordination of study protocol

Sponsor Scientific Coordinator

Filippo Belardelli, PhD

Institute of Translational Pharmacology (IFT)
National Research Council (CNR), Rome, Italy
Phone: +390649934486 Fax: +390645488257
e-mail: filippo.belardelli@ift.cnr.it
Responsible for the management of the MERCK Grant and of the scientific coordination of the entire project

Co-investigators:

Nazario Bevilacqua, MD
National Institute for Infectious Diseases “Lazzaro Spallanzani”
Via Portuense 292, 00149 Rome, Italy

Phone: 06-55170232
e-mail: nazario.bevilacqua@inmi.it
Responsible for patients enrollment and management

Nicola Vanacore, Ilaria Bacigalupo, Flavia Lombardo and Antonio Ancidoni
National Centre for Disease Prevention and Health Promotion
Istituto Superiore di Sanità
Viale Regina Elena, 299
Roma, Italy
Phone: +390649904243
e-mail: nicola.vanacore@iss.it; ilaria.bacigalupo@iss.it; flavia.lombardo@iss.it; antonio.ancidoni@guest.iss.it
Responsible for Statistical design, data management and analysis

Eleonora Aricò and **Luciano Castiello**

FaBioCell, Core Facilities
Istituto Superiore di Sanità
Phone: +390649902414
e-mail: Eleonora.arico@iss.it; Luciano.castiello@iss.it
Responsible for study design, protocol writing and for the exploratory analysis on IFN signaling

Laura Bracci

Department of Oncology and Molecular Medicine
Istituto Superiore di Sanità
Phone: +390649902474
e-mail: laura.bracci@iss.it
Participation to protocol writing and responsible for inflammatory cytokine analysis

Francesca Urbani

Department of Oncology and Molecular Medicine
Istituto Superiore di Sanità
Phone: +390649903698
e-mail: francesca.urbani@iss.it
Responsible for CRF design, participation to protocol writing and responsible, together with Iole Macchia, of the exploratory analysis on cellular immunomonitoring

Roberto Nisini and **Anna Rita Ciccaglione**

Department of Infectious Diseases
Istituto Superiore di Sanità
Phone: +390649902659, +390649903233
e-mail: roberto.nisini@iss.it; annarita.ciccaglione@iss.it
Responsible for SARS-CoV 2-Specific Binding Antibody analysis

Ombretta Papa,

Special Units for Regional Continued Care (USCAR)
e-mail: dott.papa@outlook.com
Participating in the enrollment and management of non-hospitalized patients. Responsible for the establishment of the network of family doctors for the early detection of non-hospitalized patients

Concetta Castilletti and **Maria R. Capobianchi**

Laboratory of Virology
National Institute for Infectious Diseases "Lazzaro Spallanzani"
Responsible for diagnostic analyses of COVID-19 patients enrolled at INMI

Antonino Di Caro, Stefania Carrara and Donatella Vincenti

Microbiology Laboratory and Infectious Diseases Biobank
National Institute for Infectious Diseases "Lazzaro Spallanzani"
e-mail: antonino.dicaro@inmi.it, stefania.carrara@inmi.it, donatella.vincenti@inmi.it
Responsible for INMI BioBank processing and storage of biological samples

Silvia Murachelli

Pharmacy Unit
National Institute for Infectious Diseases "Lazzaro Spallanzani"
e-mail: silvia.murachelli@inmi.it
Responsible for experimental drug storage at INMI pharmacy

Other laboratories involved:

Synlab Lazio srl
Via San paolo Dei Cavalieri 20 00159 Roma
Tel.: 06 438 6280
Email: mycete@synlab.it
Responsible for SARS-CoV-2 RT-PCR analysis on nasopharyngeal swabs

Clinical research organization

FullCro srl
Via Ignazio Guidi 3, 00147 Roma
Tel. +39.06.58.30.03.26, Fax +39.06.58.30.03.09
Email: info@fullcro.org

Administrative support:

Matilde Paggiolu, **Giuseppina Ozzella** and **Pamela Papa**
Institute of Translational Pharmacology (IFT)
National Research Council (CNR)
e-mail: matilde.paggiolu@ift.cnr.it, giuseppina.ozzella@ift.cnr.it, pamela.papa@ift.cnr.it
Administrative clinical research support on inter-institutional agreements, material transfer agreements, institutional tenders.

………………………………………………

This is an investigator-initiated study. The Steering Committee will take responsibility for study design and data analysis and will operate actions necessary to guarantee that the trial is conducted in accordance with procedures described in this document and good clinical practice. The study is partially funded by Merck. Merck has no role in study design, data collection, management, analysis, data interpretation, manuscript writing, or in the decision to submit manuscripts for publication.

The Steering committee will include at least one representative from all units participating to the study and will be chaired by the Scientific Coordinator of the study (Filippo Belardelli) with the cooperation of a coordination team (Giuseppe Sconocchia, Emanuele Nicastri, Ombretta Papa, Nicola Vanacore, Eleonora Aricò, Luciano Castiello). The Steering committee will oversee all the aspects of the project's life: decision about safety, decision for stopping rule, diagnostics issues, capacity development, financial, schedule, partnership, dissemination and exploitation. The Steering committee will hold at least one meeting a week on teleconference. In addition, extraordinary sessions will be held in case of critical issues.

1. Synopsis

1.1 BACKGROUND

The rapid and devastating outbreak of Coronavirus disease 2019 (COVID-19) pandemic highlighted the urgent need of developing therapeutic options to control or prevent virus spreading. In this regard, priority should be given to the repurposing of existing antiviral agents, thus shortening the timelines needed for clinical experimentation while exploiting the clinical experience with other viral infections (1). Among the many drugs under evaluation all over the world, Interferon (IFN)-α and β stirred renewed interest against COVID-19 and are presently being evaluated in clinical trials at different dosages and by different delivery systems, either as monotherapy or in combination with other compounds. Notably, IFN-β proved effective in alleviating COVID-19 symptoms when used in combination with lopinavir and ritonavir (2) and in reducing mortality when combined with hydroxychloroquine and other antivirals (3).

IFN-α and β, thereafter referred to as type I IFN (IFN-I), are cytokines with a long record of clinical use in patients with infectious disease (4), multiple sclerosis, and cancer (5). They are pleiotropic factors endowed with multiple activities, including both a broad spectrum antiviral activity and a remarkable immunoregulatory function (6). IFN-I are expressed at very low levels under basal physiological conditions, while they are generally abundantly produced in response to virus infections, when they play a crucial role in limiting viral replication and spread (7). In fact, many viruses, including Coronaviruses, evolved evasion strategies to counteract IFN-I system activation (8,9).

An ensemble of studies, some of them carried out in the proponents' laboratories, have revealed that in addition to the antiviral activity, optimally achieved in the first phase of infection, IFN-I exhibit important immunoregulatory effects, including the increase of neutralizing antibodies and the induction of both innate and adaptive cellular immunity (10–15).

While the majority of SARS-CoV 2 infected individuals are capable of clearing the virus solely with their own immune response, approximately 20% develops severe COVID-19. Notably, at higher risk of severe COVID-19 are males, people aged >65 years and/or showing some comorbidities (like hypertension and diabetes). An age-related impairment of endogenous IFN-I induction in response to viral infection has been described (16). Data on animal models on SARS-CoV (17,18) and data emerging from COVID-19 pandemic (19–22) point out to endogenous IFN-I system as a key player

to control early phases of viral replication and prevent disease progression. Moreover, delayed IFN-I signaling activation can contribute to the exacerbation of SARS-CoV hyperinflammation and subsequent viral pathogenesis (20,23,24).

In the light of these considerations and evidences, we hypothesize that elderly patients will greatly benefit from a short term IFN-β1a administration at the earliest time of SARS-CoV 2 diagnosis, thus compensating the insufficient or impaired endogenous IFN-I production.

In these patients, the antiviral and immunomodulatory effects of this cytokine could be efficiently exploited against COVID-19 through a short-term, discontinuous treatment with IFN-β1a in the early phases of infection, thus minimizing the relevant side effects (refractoriness and toxicity) associated to IFN continuous treatment schedules.

1.2 Objectives

This trial aims at exploring the efficacy of IFN-β1a in reducing the risk of SARS-CoV 2 recently infected elderly patients to progress towards severe COVID-19. In particular, this study will evaluate the consequences of a low and discontinuous use of IFN-β1a in the early phase of infection, and to exploit its immune activating properties in addition to its antiviral effects. Such regimen is expected to prevent any toxicity and refractoriness phenomena often occurring during IFN-I chronic administration.

Primary Objective of the study is to evaluate the role of IFN-β1a in reducing the disease progression in treated patients versus control group.

Secondary Objectives of the study are: 1) to assess the reduction in ICU admission in patients treated with IFN versus control group; 2) to assess the reduction in number of deaths in IFN compared to control group; 3) to evaluate the increase in proportion of participants returning to negative SARS-CoV 2 RT-PCR in IFN-treated *versus* control group at Day 14 and Day 28; 4) To assess the increase in SARS-CoV 2-Specific Antibody Titers in IFN-treated compared to control group; 5) to assess the safety of IFN-treated patients.

1.3 Methodology

Randomized, Open-Label, Controlled, Phase II Study. The study plans to enroll 60 patients: 40 in the IFN-β1a arm, 20 in the control arm, according to a 2:1 - treated: untreated ratio. Treatment plan foresees 4 subcutaneous injections of 3MIU of IFN-β1a, to be given at day 1, 3, 7 and 10 in addition to standard of care. Patients will be monitored and disease progression will be evaluated by means of the National Early Warning Score (NEWS2).

1.4 Expected results

Data emerging from the ongoing pandemic show that the management of advanced stage COVID-19 is mostly critical for elderly patients. This study is expected to provide information about the efficacy of a timely administration of IFN-β to elderly patients in achieving a more efficient control of SARS-CoV 2 infection, thus preventing the progression towards severe forms of the disease. The results of this study will provide a treatment option for high-risk elderly patients experiencing mild symptoms, for which no approved therapy is available so far (besides support therapy and a strict clinical monitoring).

The proposed treatment, upon demonstration of efficacy and safety, could be administered not only to hospitalized patients, but also during isolation at home or in long-stay residential care homes (LSRCHs), with the support of the territorial medical units. Therefore, this treatment protocol will represent an important tool to protect the elderly population in every pandemic scenario that will occur in the near future.

2. Background

The rapid and devastating outbreak of Coronavirus disease 2019 (COVID-19) pandemic and the lack of approved treatments for any human coronavirus (CoV) infection highlighted the urgent need of developing therapeutic options to control or prevent virus spreading. Several options can be envisaged ranging from prophylactic vaccine to targeted antiviral drugs. However, new interventions are likely to require months to years to be developed, and priority is being given to the repurposing of existing antiviral agents (1). Since COVID-19 outbreak, more than 3000 clinical trials have been authorized to identify the drugs or drug combinations capable of attenuating the

virulence of the disease (25). Some of these trials include the use of type I Interferons (IFN-I), mainly α and β, alone or in combination with other compounds. Interestingly, a randomized clinical trial testing the combination of Lopinavir, Ritonavir plus IFN-β in COVID-19 patients showed that only the triple combination was effective in alleviating symptoms and shortening the duration of viral shedding and hospitalization (2). A significant reduction of mortality was observed when IFN-β was administered together with hydroxychloroquine and other antivirals (3). Notably, data suggest that the timing of IFN therapy during SARS-CoV 2 infection can determine treatment efficacy and clinical outcome (26).

IFN-I were first discovered and characterized more than 60 years ago as antiviral substances produced by influenza virus-infected cells, capable of markedly inhibiting viral replication in target cells (27). These cytokines were the firsts to be cloned and extensively used in patients with some viral diseases (28) and cancer (IFN-α) (5). IFN-I are pleiotropic factors endowed with multiple activities, including both a broad-spectrum antiviral activity (27,28) and a remarkable immunoregulatory function (6). The antiviral activity of IFN-I has been extensively exploited for the treatment of viral chronic infections (28) Nevertheless, as highlighted by the long clinical records of IFN-I use, caution is required in terms of route, timing and dose of administration to balance clinical efficacy and side effects.

As many other viruses, Coronaviruses have developed multiple mechanisms to prevent IFN-I induction and subsequent signaling (29), particularly during the early phase of infection, ultimately leading to a dysregulated immune response and increased immunopathogenesis (20,30,31). Diminished levels of IFN-I have been detected in patients during the course of SARS and MERS (32–34). Similar results were also achieved with aged macaques infected with SARS-CoV, that exhibited considerably lower levels of IFN-β and a more severe pathology than young animals (17). Interestingly, when the deficiency in IFN-I production in CoV-infected macaques was remedied by IFN-α2 treatment in combination with ribavirin, lower levels of systemic (serum) and local (lung) proinflammatory markers were observed, in addition to fewer viral genome copies and less severe histopathological changes in the lungs (18). More relevantly, the results of a recent work clearly showed an impaired IFN-I signaling, associated with persistent blood virus load and an exacerbated inflammatory response in patients with severe COVID-19 (35). Impaired IFN-I response was also observed in young men experiencing severe COVID-19, in which a loss-of function genetic mutation in Toll Like Receptor 7 caused impaired IFN-I response (21). Overall, these observations outline the

critical role of IFN-I in both protective and pathogenic events during CoV infections, thus strengthening the need of fine tuning the IFN-I signaling with respect to the kinetics of CoV replication for an optimal protective response.

3. Rationale

In the light of the current information on SARS-CoV 2 pathogenesis, we speculate that the majority of SARS-CoV 2- infected patients are capable of clearing the virus by means of their effective endogenous IFN-I system and do not require hospitalization. We assume that in a minority of people a defective IFN-I system may favor SARS-CoV 2 spread, eventually causing the development of severe forms of COVID-19 and dismal prognosis. People aged >65 years, for which an impairment of IFN-I induction in response to viral infection has been documented (16,36,37), are at higher risk of severe COVID-19 (38).

In these patients, a delayed IFN-I response and the loss of viral control might contribute in early phases of infection to disease outcome. Data suggest that the IFN-β subtype appears to be the most suited for COVID-19 treatment (39). Thus, we hypothesize that elderly patients will greatly benefit from a short term IFN-I administration at the earliest time of SARS-CoV 2 infection, thus compensating the insufficient or impaired endogenous IFN-I production and preventing COVID-19 progression to severe forms of disease. In light of its immunomodulatory properties, IFN-β administered at the early phases of infection can represent a valuable tool to enhance humoral and cellular immunity in addition to its direct antiviral treatment restricting early viral spread, thus halting virus replication and preventing the progression towards severe forms of disease.

4. Impact for the National Health System

Italy was the first European country to experience COVID-19 pandemic, when the information about viral pathogenesis and therapeutic options were scarcely available. Moreover, Italian demographic structure, with a high percentage of population above 65 years of age, greatly affected the outcome and the death toll of the first epidemic wave. In fact, data show that not only sex and comorbidities, but also age increases the risk of developing severe COVID-19 (38,40) needing hospitalization and intensive care support. Since the first case, recorded in Italy on February 21th, COVID-19 represented a big challenge for the Italian National Health System, which underwent an increasing pressure until

restriction measures were undertaken to avoid its collapse. However, the interruption of non-essential economic and social activities has a serious impact on global economy and people quality of life in the long term. For elderly people, isolation can result not only in increased risks of cardiovascular, autoimmune and neurocognitive disorders, but also induce or exacerbate mental health problems, such as depression and anxiety. The introduction of a new phase, in which the restriction measures were gradually released and economic activities restarted, required some strategies to be undertaken to keep an acceptable risk for all population. A reinforced surveillance system was developed and is currently in use to ensure a prompt diagnosis of new cases. Nevertheless, it is urgent to develop and test new treatment options that can be administered during the early infection to reduce viral shedding, and consequent contagion, and to hamper disease progression toward severe forms, thus diminishing the impact on the National Health System.

In this trial, particular attention is given to aged patients with a recent diagnosis of COVID-19 in the presence of mild symptoms. In these patients, a strict medical control during home isolation, or a precautionary hospitalization are both appropriate choices, to monitor the possible rapid evolution of the infection. However, no therapeutic regimen specifically designed for these patients is available. Therefore, the risk of developing severe forms of the disease requiring intensive care or ending in fatalities is still high.

This trial will test the efficacy of IFN-β administered to aged patients during the early phase of the infection, in limiting viral replication and preventing the evolution of COVID-19 towards severe and critical diseases. Individual infectivity is directly associated with disease severity and time of viral shedding. Moreover, preventing severe COVID-19 will directly reduce lethality and will immediately mitigate the hospitals overworking, thus overall reducing the potential impact of COVID-19 on the National Health System.

5. Objectives of the study

This trial aims at exploring the use of IFN-β1a in SARS-CoV 2 newly diagnosed elderly patients with increased risk of developing severe COVID-19. In particular, this study will evaluate low-dose and discontinuous use of IFN-β1a in the early phase of infection, in order to exploit not only its antiviral,

but also its immune activating and anti-inflammatory properties. Such regimen should avoid any toxicity and refractoriness phenomena often occurring during IFN-I chronic administration.

5.1 Primary Objective

Primary Objective of the study is to evaluate the reduction in disease progression in patients treated with IFN versus control group within 28 days.

5.1.1 Primary endpoint and outcome

Primary endpoint of the study is the proportion of patients experiencing a disease progression, during at least 5 days, according to the National Early Warning Score **(NEWS2).** The **NEWS2** score is a standardized approach aimed at promptly detecting signs of clinical deterioration in acutely ill patients and establishing the potential need for higher level of care. It is based on the evaluation of vital signs including respiratory rate, oxygen saturation, temperature, blood pressure, pulse/heart rate, AVPU response. The resulting observations, compared to a normal range, are combined in a single composite "alarm" score. Any other clinical sign clearly indicating a disease worsening will be considered as disease progression.

5.2 Secondary Objectives and Endpoints

The following table 1 contains the secondary objectives and endpoint of the study

Objective	Endpoint
1) To assess the reduction in ICU admission in patients treated with IFN versus control group within 28 days of randomization	ICU-free days at 28 days (Day 1 through Day 28)
2) To assess the reduction in number of deaths in IFN compared to control group (day 28)	All-cause mortality (Day 1 through Day 28)
3) To evaluate the increase in proportion of participants returning to negative SARS-CoV 2 RT-PCR in IFN-treated versus control group at Day 14 and Day 28	Negative SARS-CoV 2 RT-PCR at day 14 post-randomization<br>Negative SARS-CoV 2 RT-PCR at day 28 post-randomization

4) To assess the increase in SARS-CoV 2-Specific Binding Antibody Titers in IFN compared to control group (day 28)	Change from Baseline in SARS-CoV 2-Specific Binding Antibody Titers at day 14 and 28
5) To assess the safety of IFN-treated patients versus control group	Incidence of adverse events

For secondary endpoints, more detailed descriptions follow:

1) ICU-free days at 28 days will be calculated as the number of days a patient is not in an ICU. Time Frame will be: Admission (day 0) to 28 days after admission (day 28). In case of death, it will be counted as 0 day;

2) All-cause mortality will be: total number of death events occurring within day 0 and day 28;

3) Negative SARS-CoV 2 RT-PCR is defined as an undetectable presence of SARS-CoV 2 genes, as determined by PCR on an adequate sampling of upper respiratory tract.

4) Change from Baseline in SARS-CoV 2-Specific Binding Antibody Titers is defined as the difference in anti-SARS-CoV 2-specific antibody levels measured at day 28 versus day 0;

5) Details on safety event are described in paragraph 9.4

5.3 Exploratory Endpoints

Exploratory studies will be also performed on blood samples collected before and after treatment to assess:

- IFN-I signaling activation
- Cellular immune monitoring
- Systemic inflammatory markers

5.3.1 IFN-I Signaling

Pioneer studies in animal models showed that the complete absence of IFN-I signalling, by deletion of IFN-I receptor, enhanced mice susceptibility and mortality from several viral infections (7). IFN-I signalling downregulation may occur during viral infections as a consequence of viral-specific evasion mechanisms that Coronaviruses mainly establish during the early phase of infection (29,31). Diminished levels of IFN-I or Interferon Stimulated Genes (ISG) expression have been detected in the peripheral blood mononuclear cells of SARS and MERS patients (32,33). More relevantly, the

results of a very recent work clearly showed an impaired IFN-I signaling, associated with persistent blood virus load and an exacerbated inflammatory response in patients with severe COVID-19 (35). A diminished level of endogenous IFN-I activation and signalling may also occur as a consequence of aging, as reported in several *in vitro* and *in vivo* settings (17,41). In light of these considerations, the level of expression of selected ISG will be analysed in patients PBMC as surrogate markers of IFN-I signalling activation. Samples will be collected before, during and after the completion of IFN-β1a treatment in order to assess 1) possible correlations between IFN-I activation status and patient clinical outcome *per se*; 2) treatment-induced modifications of IFN-I signalling activation possibly associated with clinical improvement.

5.3.2 Cellular Immune-Monitoring

A decrease in peripheral lymphocyte count (with lower frequencies and absolute counts of CD3, CD4, CD8 T cells as well as of NK subsets) and an inflammatory cytokine storm may be the main reasons for rapid disease progression and poor treatment response in severe COVID-19. The neutrophil-to-CD8+ T cell ratio and the neutrophil-to-lymphocyte ratio were identified as prognostic factors affecting the prognosis for severe COVID-19 (42). Besides quantitative alteration, T cell maturation status was found to be modified since the percentage of naïve helper T cells increases and memory helper T cells decreases in severe cases. Patients with COVID-19 have also low levels of regulatory T cells, showing damaged features in severe cases (43). In general, COVID-19 patients show marked T cell activation, senescence, exhaustion and skewing towards Th17, if compared to healthy subjects (44).
The innovative technology MFC will help in elucidating the immunomodulatory *in vivo* effect of IFN β1a treatment. Leukocyte subpopulation frequency, activation status and functionality will be explored in pre- and post-treatment patients' blood samples. MFC results will be correlated with clinical outcome in order to identify potential peripheral immune markers of response to treatment.

5.3.3 Systemic inflammation

It was reported that in some COVID-19 patients, the immune response elicited against SARS-CoV 2 results in an increase in systemic inflammatory cytokines, which may eventually progress to a "cytokine storm," followed by multi-organ system dysfunction (45). In fact, some of the severe manifestations of COVID-19 are linked to the excess of circulating pro-inflammatory cytokines: acute respiratory distress syndrome, thromboembolic diseases such as acute ischemic strokes caused by

large vessel occlusion and myocardial infarction, encephalitis, acute kidney injury, and vasculitis (46). The chronic activation of pro-inflammatory pathways documented in the elderly, especially men, and named "inflamm-aging", represents a risk factor *per se* for the development of COVID-19 complications (47).

We believe the restoration of a functional IFN-I response, through the administration of IFN-β1a during the early phase of SARS-CoV 2 infection, may affect systemic hyper inflammation both directly, by means of the immunomodulatory properties of the cytokines, and indirectly as an effect of reduced SARS-CoV 2 replication.

The level of inflammatory markers known to have a prognostic role in COVID-19 progression, such as IL-6, CRP, TNF-α (48) together with some endothelial cell adhesion molecules whose expression levels correlate with COVID-19 severity (FKN, VCAM-1, ICAM-1, VAP-1 (49)), will be analysed in the blood collected from IFN and control arm before and 10 days after enrolment. Data will be integrated with the results of routine lab analysis on coagulations factors (Fibrinogen, D-dimers) also involved in COVID-19 pathogenesis. The comparative analysis between groups will address treatment-induced modulations and possible correlation with clinical outcome.

5.4 Statistical hypothesis

The trial power has been calculated by the ISS group. The study was powered to independently assess a potential benefit of IFN-β1a compared with control arm (no specific antiviral treatment besides standard of care) on rate of progression of NEWS2 score lasting more than 5 days.

Sample size was calculated according to the primary endpoint of the study. In particular, the sample size calculation is based on the assumptions of an at least 35% difference in the percentage of patients undergoing disease progression between IFN-β1a and control arm. A sample size of 60 patients total (40 in the IFN-β1a-treated arm and 20 in the control arm, according to a 2:1 randomization ratio) will be needed to provide 80% power at significance level of 5% to detect the difference of patients undergoing disease progression between a group 1 proportion of 0.15 (IFN-β1a + standard of care) and a group 2 proportion of 0.50 (standard of care).

Sample Size: ANTIICIPATE trial

Two-sided significance level (1-α)	95
Power (1-β, % chance of detecting)	80
Ratio of sample size, Unexposed/Exposed	0.5
Percent of Unexposed with Outcome	50
Percent of Exposed with Outcome	15
Risk Ratio	0.3
Risk difference	-35

	**Kelsey**	**Fleiss**	**Fleiss (CC)**
Sample Size - Exposed	39	40	48
Sample Size-Unexposed	20	20	24
Total sample size	59	**60**	72

6. Study design

Randomized, Open-Label, Controlled, Phase II Study. Patients, who satisfy all inclusion criteria and no exclusion criteria, will be randomly assigned to one of the two treatment groups in a ratio 2:1. Randomization will be stratified by gender. Stratified randomization will balance the presence of male and female in both study arms. The planned study duration is 12 months including study set up, enrollment, follow up and data analysis as indicated in Appendix 3.

7. Study Population

Male and female adults aged 65 years or older with newly diagnosed mild COVID-19 are eligible for the study.

7.1 Case definition

For the purpose of the study, the following definition is applied: a case of COVID-19 is a person with detectable SARS-CoV 2 genes, as determined by PCR on an adequate sampling of upper respiratory tract.

7.2. Criteria for eligibility

7.2.1 Inclusion criteria

- ≥ 65 years of age at time of enrolment;
- Laboratory-confirmed SARS-CoV 2 infection as determined by PCR, in any specimen < 72 hours prior to randomization;
- Subject (or legally authorized representative) provides written informed consent prior to initiation of any study procedures;
- Understands and agrees to comply with planned study procedures;
- Agrees to the collection of nasopharyngeal swabs and venous blood samples per protocol;
- Being symptomatic for less than 7 days before starting therapy;
- NEWS2 score ≤2

7.2.2 Exclusion criteria

- Hospitalized patients with illness of any duration, and at least one of the following:
  -Clinical assessment (evidence of rales/crackles on exam) AND SpO2 ≤ 94% on room air at rest or after walking test,
  OR
  -Acute respiratory failure requiring mechanical ventilation and/or supplemental oxygen;
- Patients currently using IFN-beta (e.g., multiple sclerosis patients);
- Patients undergoing chemotherapy or other immunosuppressive treatments
- Patients with chronic kidney diseases;
- Known allergy or hypersensitivity to IFN (including asthma);
- Any autoimmune disease (resulting from patient anamnesis);
- Patients with signs of dementia or neurocognitive disorders;

- Patients with current severe depression and/or suicidal ideations;
- Being concurrently involved in another clinical trial;
- HIV infection (based on the anamnesis);
- Use of any antiretroviral medication;
- Impaired renal function (eGFR calculated by CKD-EPI Creatinine equation < 30 ml/min);
- Presence of other severe diseases impairing life expectancy (e.g. patients are not expected to survive 28 days given their pre-existing medical condition);
- Any physical or psychological impediment in a patient that could let the investigator to suspect his/her poor compliance;
- Lack or withdrawal of informed consent

7.3 Recruitment strategy

The management of elderly patients with COVID-19 needs to take into consideration the presence of comorbidities that increases their fatality risk, but it is also affected by the epidemiological situation of SARS-CoV 2 infection (see Feasibility section). Our study plans to enroll either hospitalized and non-hospitalized newly diagnosed COVID-19 patients, as well as patients hosted in long-stay residential care homes.

The Special Unit for regional continued care (USCAR), having the role of early detecting clusters of infection within Regione Lazio, will be responsible for screening and enrolling eligible patients that after SARS-CoV 2 positivity notification are not hospitalized, but remain in isolation at home or in a long-stay residential care homes. When dealing with patients older than 65, USCAR will be responsible of informing the patient of the current study, of having the Informed Consent signed and of collecting the blood sample to assess eligibility criteria. After the enrolment, the patient will be followed by a dedicated USCAR team that will: i) give to the patient/family-caregiver the kit of devices for home monitoring (i.e., 1 pulse oxymeter, 1 digital sphygmomanometer, 1 thermometer), ii) perform training for the use of devices, iii) perform treatments and collect samples for monitoring patients according to the timeline described in Appendix 2. The USCAR team will receive daily updates from non-hospitalized patients to determine their NEWS2 score values.

Patients that, at discretion of the general practitioner, are directed to Spallanzani Hospital for hospitalization, will be there assessed for inclusion/exclusion criteria and, in case of eligibility, enrolled in the study. Patients will be monitored according to standard hospital protocol in addition to the timeline described in Appendix 2.

8. Intervention

8.1 Experimental Drug and justification for dose

Rebif® (interferon beta-1a) is a disease-modifying drug used to treat relapsing forms of multiple sclerosis (MS) and is similar to the IFN-beta protein produced by the human body. It was approved in Europe in 1998 and it is used in more than 90 countries worldwide. While current posology of Rebif in MS (12 MIU 3 times/week) is capable of balancing the neural inflammation typical of MS, the dosing and schedule of Rebif® administration in this study were selected by taking into consideration some features of IFN-I, emerged from many years of clinical use of these cytokines. In fact, several clinical studies reported that an Interferon-induced immune adjuvant activity could be observed already after the administration of intermittent low doses of the cytokine in both cancer and antiviral settings (15,50–52). Instead, the continuous stimulation of IFN-I signaling, exerted by high serum levels of the cytokine, can result in diminished treatment efficacy due to the emergence of refractoriness phenomena caused by receptor internalization/degradation as well as the rapid induction of UBP43 and SOCS negative regulators (4), immunosuppression and can also result in relevant side effects.

With the aim to tailor the treatment schedule to the early phase of SARS-CoV 2 infection in elderly patients, we selected 3 MIU of IFN-β1a as a dose expected to exploit IFN-mediated antiviral and immunomodulatory properties of the cytokine without causing relevant toxicity or inducing refractoriness phenomena (53).

8.2 Treatment arms

Control arm. No specific antiviral treatment besides standard of care.

Treatment arm. 11ug (3MIU) of IFN-β1a will be injected subcutaneously at day 1, 3, 7, and 10 in addition to standard of care. The drug solution, contained in a pre-filled cartridge, will be injected by means of the RebiSmart electronic injection device, as described in Appendix 6.

8.3 Standard patients monitoring

Patients will be daily evaluated for body temperature, respiratory rate, oxygen saturation, blood pressure, pulse/heart rate and AVPU response. The NEWS2 score will be then calculated following the table 2. Additional measurements are allowed whenever any sign of disease progression appears. In case of multiple measurements within a day, the highest score will be considered for patient assessment.

Table 2. NEWS2 Score

Chart 1: The NEWS scoring system

Physiological parameter	Score						
	3	2	1	0	1	2	3
Respiration rate (per minute)	≤8		9–11	12–20		21–24	≥25
SpO_2 Scale 1 (%)	≤91	92–93	94–95	≥96			
SpO_2 Scale 2 (%)	≤83	84–85	86–87	88–92 ≥93 on air	93–94 on oxygen	95–96 on oxygen	≥97 on oxygen
Air or oxygen?		Oxygen		Air			
Systolic blood pressure (mmHg)	≤90	91–100	101–110	111–219			≥220
Pulse (per minute)	≤40		41–50	51–90	91–110	111–130	≥131
Consciousness				Alert			CVPU
Temperature (°C)	≤35.0		35.1–36.0	36.1–38.0	38.1–39.0	≥39.1	

For non-hospitalized patients, measurements will be auto-performed by the patient either assessed by a caregiver or a family member. Training on how to use the provided devices will be performed by USCAR unit at T1, written instructions will be also provided, and additional help will be given

upon request by phone or videocall. Measurements will be recorded on the clinical diary that will be provided (Appendix 5). Patients will be contacted daily by USCAR dedicated unit and will communicate by phone their health status that will be registered on a dedicated clinical records form (Appendix 5). USCAR unit approaching COVID-19 patients will use personal protective equipment including a FFP3 (or FFP2) mask, gloves, gown and googles. FFP3 will be used always in case of any procedure on respiratory tract (including nasopharyngeal swab).

Hospitalized patients can be discharged from the hospital considering the ongoing national and regional recommendations to discharge COVID-19 patient at home. The USCAR unit will then responsible of continuing follow up of the patient according to the timeline described in Appendix 2.

8.4 Other therapies allowed

Patients will not receive any other antiviral treatment, unless considered needed by the physician. All other treatments including anti-hypertensive drugs, medications for diabetes (insulin and oral drugs), antibiotics, hormone therapy can be provided to patients of both groups according to medical judgments. Patients should not receive nonsteroidal anti-inflammatory drugs apart from paracetamol if needed.

Any previous and concomitant medication will be recorded along with:

- Reason for use
- Dates of administration including start and end dates
- Dosage information including dose and frequency

8.5 Safety monitoring and individual stopping rules

Any sign or symptom associated to drug adverse events will be daily reported.

Progressing patients which are in need of oxygen support will be maintained in the trial for follow up purposes, but treatment will be discontinued (Appendix 1). Progressing patients will receive standard of care or additional treatment at the physician discretion.

Another stopping rule includes drug related adverse events grade ≥ 3 according to Common Terminology Criteria for Adverse Events (CTCAE) version 5.0.

For progressing patients, we will record the admission to the intensive care unit, days in ICU, and the disease outcome (either survivor or non-survivors) by using the Regional surveillance systems.

Patients are free to withdraw from participation in the study at any time upon request, without any consequence. Patients should be listed as having withdrawn consent only when they no longer wish to participate in the study and no longer authorize the Investigators to make efforts to continue to obtain their outcome data. Every effort should be made to encourage patients to remain in the study for the duration of their planned outcome assessments. Patients should be educated on the continued scientific importance of their data, even if they discontinue the study. In the case of a patients becoming lost to follow-up, attempts to contact the patient should be made and documented in the patient's medical records.

9. Methods

9.1 Randomization

Sixty patients will be randomized 2:1 to receive IFN-β1a or control arm. Eligible patients will be randomised (no later than 36 h after enrolment) by means of a computerized central randomization system. All patients will receive a unique patient identification number at enrolling visit when signing the informed consent and before any study procedures are performed. This number must remain constant throughout the entire study.

ISS will prepare a randomization list by using a validated software and the list will be managed by the CRO. The randomization of patients will be closed when 60 patients have been randomized. The randomization will be stratified by sex; for each stratum a sequence of treatments randomly permuted in blocks of variable length (3 or 6) will be generated.

9.2 Blinding

This is an open-label study. After the randomization, patient will be notified whether will receive or not the experimental drug.

9.3 Electronic case report form

Patients' data will be recorded in an *ad hoc* online database. The Electronic case report form will be provided by a Clinical Research Organization and implemented according to the study design. An example of the information to be recorded in the e-CRF is provided as Appendix 4.

9.4. Safety Criteria Evaluation

9.4.1 Safety profile

Subjects participating in this trial who received at least one dose of the trial medication are considered to be included in the safety population (full analysis population). Safety population not include subjects who drop out prior to receiving any treatment. Data on safety profile, nature, incidence and severity of adverse events (AEs) and serious adverse events (SAEs) will be collected as detailed in both this section of the protocol and in the AE/SAE section of the CRF. Any reason for drug interruption, reduction and discontinuation will be collected. Toxicities will be graded using NCI Common Terminology Criteria for adverse Events (CTCAE) version 5. The investigator is responsible for detecting, documenting and reporting AEs and SAEs, according to the criteria defined in this protocol.

The safety profile of experimental drug (i.e., IFN-β1a, Rebif®) has been well established. Below are the very common and common adverse reactions as reported in the Summary of Product Characteristic 2010:

**Very common (≥1/10)**	**Common (≥1/100 to <1/10)**
Neutropenia, lymphopenia, leukopenia, thrombocytopenia, anaemia	Diarrhoea, vomiting, nausea
Asymptomatic transaminase increase	Severe elevations in transaminases
Headache	Pruritus, rash, erythematous rash, maculo-papular rash, alopecia
**Injection site inflammation, injection site reaction, influenza-like symptoms**	Myalgia, arthralgia
	Depression, insomnia
	Injection site pain, fatigue, rigors, fever

9.4.2 Adverse events (AE) and serious adverse events (SAE)

9.4.2.1 Definition of an AE

An AE is defined as any untoward medical occurrence in a patient, temporarily associated with the use of a medicinal product, whether or not it is considered related to the medicinal product. An AE can therefore be any unfavorable and unintended sign (including an abnormal laboratory finding), symptom, or disease (new or exacerbated) temporarily associated with the use of a medicinal product. Examples of an AE include:

· Significant or unexpected worsening or exacerbation of the condition/indication under study.

· Exacerbation of a chronic or intermittent pre-existing condition including either an increase in frequency and/or intensity of the condition.

· New conditions detected or diagnosed after investigational product administration even though it may have been present prior to the start of the study.

· Signs, symptoms, or the clinical sequelae of a suspected interaction.

· Signs, symptoms, or the clinical sequelae of a suspected overdose of either investigational product or a concurrent medication (overdose per se should not be reported as an AE/SAE).

9.4.2.2 Definition of a Serious Adverse Event

A serious adverse event (SAE) is defined as any untoward medical occurrence that, at any dose:

1. Results in death
2. Is life-threatening
3. Requires hospitalization or prolongation of existing hospitalization
4. Results in disability/incapacity
5. Is a congenital anomaly/birth defect
6. Is otherwise considered as medically important.

9.4.2.3 Recording of AEs and SAEs

When an AE/SAE occurs, it is the responsibility of the investigator to review all documentation (e.g. hospital progress notes, laboratory, and diagnostics reports) relative to the event. The investigator will then record all relevant information regarding an AE or SAE on the eCRF. Any AEs or SAEs occurring during the study must be documented in the subject's medical records and on the appropriate page of the eCRF. Each AE or SAE is to be recorded individually. All AEs which occur

during the course of the study should be recorded in the eCRF. Information on the AE must be recorded on a specific AE form (appendix 5).

9.4.2.4 Evaluating AEs and SAEs

9.4.2.4.1 Assessment of intensity

The investigator will make an assessment of intensity of each AE and SAE reported. In this protocol, the intensity of AEs and SAEs will be graded on a scale of 1 to 5 according the National Cancer Institute (NCI) Common Toxicity Criteria for Adverse Events (CTCAE) Version 5 and are available at https://evs.nci.nih.gov/ftp1/CTCAE/About.html .

For SAEs, the maximum intensity (or grade) will be reported in the eCRF. For non-serious AEs, each change in intensity (or grade) will be reported in the eCRF.

9.4.2.4.2 Assessment of causality

The investigator is obliged to assess the relationship between the study medical product and the occurrence of each AE/SAE and provide the assessment of causality as per instructions on the SAE form in the Investigators File.

9.4.2.4.3 Follow-Up of AEs and SAEs

After the initial AE/SAE report, the investigator is required to proactively follow each subject and provide further information on the subject's condition by any supplemental investigations as may be indicated to elucidate the nature and/or causality of the AE or SAE. AEs that are ongoing with a toxicity of Grade 3 or 4, or have a relationship to study drug that is suspected (Reasonable Possibility) will be queried for resolution at study conclusion and at approximately 30 days after the last dose of study. New or updated information will be recorded on the originally completed SAE form in the Investigator's File, with all changes signed and dated by the Investigator.

9.4.3 Regulatory reporting requirements for adverse events

The Investigator must report immediately (within 24 hours from the knowledge) to the study Sponsor any SAE, occurred during the study whether related to the investigational product or not.

The study Sponsor has the legal responsibility to promptly notify, as appropriate, both the local regulatory authority and other regulatory agencies about the safety of a product under clinical investigation. The study Sponsor will comply with the Italian regulatory requirements related to the reporting of SAEs to regulatory authorities and the Independent Ethics Committee (IEC). In particular, all the Suspected Unexpected Serious Adverse Reactions (SUSARs) that occur while on treatment and within 30 days since the last investigational drug administration, and that have a

suspected relationship to study's drug (Reasonable Possibility) will be notified with an urgency procedure to the local regulatory Agency (AIFA) and IEC with the following timelines:

- SUSARs that are considered life-threatening: notification within 7 days.
- SUSARs that are not considered life-threatening: notification within 15 days.

The notification with urgency procedure is not required for SAEs that are expected with the drugs used in the protocol, and for non-serious AEs, both expected and unexpected. For these events (expected SAEs and AEs), the CT will notify the local regulatory agency and IECs by an annual safety report.

9.5 Secondary and Exploratory endpoints

Dedicated blood samples will be collected at different time points (see APPENDIX 2: Timeline scheme) and processed at the biobank of the INMI.

9.5.1 SARS-CoV-2 Antibodies

The development of a specific humoral response will be monitored by measuring specific anti SARS-CoV-2 antibodies in the sera of patients collected at day 14 and 28 post randomization. Commercially available tests will be used to detect IgM specific for S antigen, IgG specific for the N and S antigens, and IgA specific for S antigen. Sera resulting reactive with the S antigen will be tested for the capacity of viral neutralization using standardized methods.

9.5.2 Molecular IFN-I signaling

Blood samples will be collected at T1 prior first treatment, during treatment (T3 prior second treatment) and post treatment (T14) and processed at the biobank of the INMI. Isolated PBMC will be aliquoted, submerged with RNA stabilization reagent and cryopreserved. For analysis, total RNA will be isolated and the transcriptional analysis of over 500 general immunology genes will be performed by means of Nanostring technology. Data analysis will determine the transcriptional modifications occurring during the course of IFN-β1a treatment as well as to identify molecular patterns potentially correlated with clinical outcome. Particular focus will be given to the ISG score reported to be differentially expressed among mild to severe COVID-19 (22). This exploratory analysis will be conducted by Dr Aricò and Dr Castiello, having a relevant background on IFN signaling analysis (51,54)

9.5.3 Cellular Immune monitoring

Pre-(T1 prior treatment) and post-treatment (T3 and T14) blood samples will be monitored by MFC-based assays through different antibody panels in order to analyze frequency of major leukocyte subpopulations associated with naïve/memory, co-activation and co-inhibition markers; polyfunctional properties of T cell specific response against virus antigens will be evaluated after short term in vitro culture.

Stained samples will be acquired on a Beckman Coulter CytoFlex Cytometer and analyzed by CytExpert and/or Kaluza software as well as by advanced machine learning algorithms such as FlowSOM and CITRUS (Cytobank online platform). Dr. Francesca Urbani and Dr. Iole Macchia, co-investigators at ISS unit, have long lasting experience in MFC assays and immune-monitoring (52,55,56).

9.5.4 Systemic Inflammatory markers

Pre- (T1 prior treatment) and after-treatment (T14) blood samples will be collected from Treatment and control group to monitor the levels of soluble factors involved in inflammation (e.g., cytokines and chemokines) and endothelial cell adhesion molecules. At the selected time points, plasma will be isolated from peripheral blood and cryopreserved until analysis that will be simultaneously conducted by means of specific ELISA assays. Data will be integrated with the results of routine lab tests on coagulation factors and factors involved in COVID-19 pathogenesis (CRP, IL-6, TNF-α, Fibrinogen and D-Dimer).

10. Statistical Plan

The primary analysis will be carried out on the primary endpoint on the intention-to-treat (ITT) population defined as all patients randomized receiving at least one dose of treatment.

The percentage of patients undergoing disease progression defined on rate of progression of NEWS2 score lasting more than 5 days will be calculated in two arms (IFN-β1a + standard of care vs standard of care) of the trial. For persons who died, a conservative approach will be adopted and death will be considered an event. The effect of treatment will be estimated through a logistic regression model including a dummy variable for treatment. The effect of treatment will be estimated through multivariable logistic regression model by accounting for the following

covariates: age, gender, co-morbidities. Moreover, NEWS2 score at baseline and setting of recruitment will be also considered.

All primary and secondary analyses will be carried out both on ITT population and on per-protocol population. Per-protocol population includes all subjects who were included in the ITT population that received the treatment as defined in the protocol and who completed the study with no major protocol violations.

Kaplan-Meier survival analysis and Cox proportional hazards model will be used for time-to-event data. The following covariates will be included in the Cox model: age, gender, co-morbidities. Moreover, NEWS2 score at baseline and setting of recruitment will be also considered. For the secondary endpoint ICU-free days, a competing risk model will be adopted considering death a competing event, following the method proposed by Fine and Gray (57). Moreover, the median difference will be reported.

The longitudinal secondary endpoint measured on a continuous scale (the increase in SARS-CoV 2-Specific Binding Antibody Titers in IFN compared to control group) will be analysed using a Mixed effect Model for Repeat Measure (MMRM) to estimate the difference of mean change from baseline in SARS-CoV 2-Specific Binding Antibody Titers between IFN-β1a + standard of care and standard of care at day 28. In case of data sporadically missing during the course of trial we will assume they were Missing At Random (MAR). A sensitivity analysis will be carried out by conducting the statistical test after imputing missing, including the worst-case imputation. All missing data will be imputed within treatment groups defined by randomized treatment.

Safety endpoint will be compared by a chi-squared test for discrete variables, by means of analysis of variance (ANOVA) and covariance (ANCOVA) for continuous variables or by the non-parametric Mann-Whitney test when appropriate.

Confidence intervals (95%) will be reported for all outcomes and association measures (proportions, means, Odds Ratios and HRs).

For all statistical analyses (efficacy and safety), the level of statistical significance will be kept at 0.05 with two-sided p-values. Statistical analyses and related reports will be in full compliance with ICH E9 guidance (https://www.ema.europa.eu/en/documents/scientific-guideline/ich-e-9-statistical-principles-clinical-trials-step-5_en.pdf).

11. Timing

See APPENDIX 2: Timeline scheme and APPENDIX 3: GANTT

12. Feasibility

Our study plans to enroll either hospitalized and non-hospitalized newly diagnosed COVID-19 patients, as well as patients hosted in long-stay residential care homes. The possible scenario of the Italian pandemic occurring during the conduction of the study will likely affect the proportion of patients that will be enrolled in the different settings. In fact, for patients presenting with mild illness, the decision to undertake hospitalization vs home care should be carefully evaluated to take into account patient risk of rapid deterioration, but also the burden on the health care system.
On august 11th, the ISS, together with the Ministry of Health and the Coordination of Italian regions and autonomous provinces, issued a document called "Elementi di preparazione e risposta a COVID-19 nella stagione autunno-invernale". The document, aimed at providing general elements and suggesting preparedness frameworks to strengthen the response and optimally cope with any increase in the number of new infections by SARS-CoV 2 in the autumn-winter 2020-2021 season, foresees four possible scenarios, characterized by increasing SARS-CoV 2 transmission rate and related risk of SSN collapse.
To ensure that patients' enrolment is duly completed in any of the possible scenarios, the study will count on a network of collaborating institutions directly involved in the identification and management of COVID-19 patients in Rome. A campaign will be held to inform Family doctors (Medici di Medicina Generale) and long-stay residential care homes (LSRCHs), whose collaboration will ensure the precocious identification of eligible patients throughout the urban area of Rome. The Special Unit for regional continued care (USCAR) are currently involved in the prompt identification of COVID-19 clusters within Regione Lazio. In this study, a group of physicians belonging to USCAR will be specifically trained and will be responsible for the screening, enrolling and clinical monitoring of patients kept under home isolation or hosted in long-stay residential care homes (LSRCHs).
All experts involved in the project are highly motivated, have complementary expertise and a strong background in the fields of IFN biology and infectious diseases. The CNR group, including the Sponsor Coordinator (Giuseppe Sconocchia) and Scientific Coordinator (Filippo Belardelli) of the study, has a long-lasting expertise on immunology, IFN biology and clinical studies with IFN-I. The

Istituto Nazionale per le Malattie Infettive “Lazzaro Spallanzani (clinical center) is one of the five clinical hubs for COVID-19 management in town and has a longstanding experience in multicentre clinical studies and the PI (Emanuele Nicastri) is a well-recognized clinician deeply involved in the clinical management of COVID-19 patients. The ISS group includes scientists with a background on IFN-I biology in both basic research and clinical trials (Eleonora Aricò, Laura Bracci, Luciano Castiello, Iole Macchia, Francesca Urbani) and with long record expertise on antibody response measurement (Annarita Ciccaglione, Roberto Nisini). Moreover, the Clinical Epidemiology group of the ISS (Ilaria Bacigalupo, Flavia Lombardo, Nicola Vanacore, Antonio Ancidoni) has a strong background on statistical analysis and was involved in the recent survey on COVID-19 infection in long-stay residential care homes (58) . To ensure the full feasibility and the high quality performance of the study the Sponsor finalized a contract with a CRO, highly specialised in clinical studies involving IFN-β, which will support the Sponsor Coordinator and the Scientific Coordinator with regard to specific services and for the implementation and monitoring of the entire study.

13. Good clinical practices and ethics

13.1. Good clinical practice

The study will be conducted in accordance with ethical principles founded in the Declaration of Helsinki (1964) and subsequent amendments and updates (Fortaleza, Brazil, October 2013), in the International Conference on Harmonization (ICH) for Good Clinical Practice (GCP) and in the appropriate regulatory requirements. The drug used in this trial is already registered and its toxicity profile is very well known, since it is largely used for the treatment of Multiple Sclerosis.

13.2 Ethical aspects

The entire study protocol, including informative material for the patients and modules for the informed consent, will be evaluated by The Ethics Committee (EC) of the National Institute for Infectious Diseases “Lazzaro Spallanzani”, Rome, Italy, which is the National Ethics Committee for evaluation of clinical trials on human drugs in COVID-19 patients.

The study will not start before obtaining a favorable opinion from the EC, the Competent Authority Authorization and any other authorization required by local regulation. Every intention to modify

any element of the original protocol after the first approval will be promptly notified to the EC and will be applied only after its written authorization. Investigators will be responsible for submitting any amendments to the protocol to the EC. Any modifications to the protocol, which may impact on the conduct of the study, may affect patient safety, including changes of study objectives, study design, patient population, sample size, study procedures or significant administrative aspects will require a formal amendment to the protocol. Such amendments will be agreed and approved by the Ethics Committee of the National Institute for Infectious Diseases “Lazzaro Spallanzani”, Rome, Italy, and the health authorities prior to implementation, in accordance with local regulation. Administrative changes of the protocol are minor corrections and/or clarifications that have no effect on the way the study is to be conducted. These administrative changes will be documented in a memorandum.

13.2.1 Written informed consent

The Investigators will ensure that the subject is given full and adequate oral and written information about the nature, purpose, possible risks and benefits of the study. Subjects will also be notified that they are free to discontinue from the study at any time. The subject’s signed and dated informed consent will be obtained prior to conduct any procedure specific for the study. The original signed Written Informed Consent Form will be stored, and a copy will be given to the patient.

13.2.2 Subject data protection

In order to protect the subjects’ identity, the Investigator will assign a subject identification number to each enrolled subject to be used instead of subject name when reporting all study related data and adverse events.

The Written Informed Consent Form will explain that the study data will be stored at Spallanzani Hospital maintaining confidentiality in accordance with national data legislation. However, the personal information must be available to authorized personnel of Study Sponsor (clinical monitor and auditor), Ethics Committee and Regulatory Authorities. In addition, consent to allow direct access to original medical records to ensure data verification will be obtained from the subject before participation in the study.

Enrolment log must be kept strictly confidential to enable patient identification at the site.

13.2.3 Audits and inspections

The Principal Investigator and the SC will provide all the necessary information and material to the participating centers in order to standardize all the protocol-related procedures and to avoid unexpected variability. Printed and electronic informative material (complete original protocol, informed consent modules, informative modules for patients and relatives, recruitment checklist, graphic timeline of interventions and visits, order list for physicians and nurses) will be distributed to Spallanzani Hospital and USCAR. Source data/documents must be available to inspections by the designee or Health Authorities.

13.2.4 Monitoring

The monitoring activities will be performed by a Clinical Research Organization. Clinical Monitor will perform the monitoring activities according to "Note of Guidance on Good Clinical Practice" (ICH E6 (R2), EMA/CHMP/ICH/135/1995).

The clinical monitor will maintain contacts between Investigators and Study Sponsor; furthermore, during the study the clinical monitor will verify that informed consent was obtained from all subjects, that the data were adequately documented in medical records and that the Investigators were compliant with the protocol. The clinical monitor will inform the study Sponsor and the Investigators about all detected protocol deviations, all facilities and technical Staff detected problems. The Investigators will provide direct access to source data/documents for data verification.

13.2.5 Declaration of interest

The study participants declare no financial and/or other conflicts of interest related to the study.

13.2.6 Dissemination policy

The Circ. Min. Health N° 6 of 09/02/2002 obliges each researcher who gets any results of interest to public health, to publish the results within 12 months from the end of the study. All the patients will freely agree or disagree to participate in the study in the belief that the results will be useful to improve knowledge about their pathologies, for health benefit from themselves or other patients. To respect their will and in the maximum interest of honest clinical research, the investigators agree on the need to ensure the wide publication and diffusion of their results in a consistent and

responsible way under their responsibility. The Study Coordinator is the official data owner. The Study Coordinator has the right to present methods and results of the study at public symposia and conferences. The principal publications from the trial will be in the name of Investigators with full credit assigned to all collaborating investigators and institutions.

13.3 Insurance

The study will be conducted according to the law about the study insurance agreement (DM 14 luglio 2009); *ad hoc* insurance n. A1202150060-LB has been established with Lloyd's Insurance Company S.A

14. Budget

**Materiale/Utilità**	**Costo unitario**	**Numero per paziente**	**N. pazienti**	**Quantità**	**Totale**
Costi coordinamento medico	75000				75000
Rebif/Rebismart	0	1	60	60	0
Servizi svolti da CRO					60000
Saturimetro	25	1	30	30	750
Sfingomanometro	20	1	30	30	600
DPI completo FFP3	10	10	30	300	3000
Assistenza domiciliare	400	1	30	30	12000
Teleassistenza	10	28	30	840	8400
Analisi del sangue	40	7	60	420	16800
Test Sars-CoV2	85	3	60	180	15300
Test sierologico	60	3	60	180	10800
Markers infiammatori	100	2	60	120	12000
Ddimero	6	2	60	120	720
Proteina C Reattiva	6	2	60	120	720
Systemic immune profiling	356	3	60	180	64080
RT-PCR per IFN signaling	280	2	60	120	33600
Costi processamento campioni biologici	30	7	60	420	12600
Assicurazione				1	9500
Costi etichettatura e gestione farmaco				1	5000
Costi generali per struttura coordinatrice					47730
Medical Writing and Statistical Data Analysis (all endpoints)					50000
**Totale**					**438600**

The study is co-funded by Merck Healthcare KGaA with a support equal to 40% of total costs.

15. Institutions agreement

The Study Sponsor will submit in the Clinical Trials all the documentation required by law to AIFA, as the Competent Authority and to Ethics Committee within a week after approval. Also, the Study Sponsor will comply in all respects with the standards of Good Clinical Practice, as defined in the

"Note of Guidance on Good Clinical Practice (CPMP/ICH 135/95)" and related Guidelines, as well as with all applicable regulatory requirements including national drug law and data protection law.
A collaboration agreement between all the Institutions involved in the study (CNR, INMI and ISS) will be signed before the enrollment of the first patient.

16. Participating Centers

- IFT, CNR, Rome;
- ISS, Rome
- INMI, Rome
- Synlab srl
- FullCro srl

17. Publications and data properties

Clinical trial data are considered the property of the investigators involved. Publications generated from the study will be sent to peer-reviewed international journals. The name and order of the authors will be decided by the working group.

18. References

1. Li G, De Clercq E. Therapeutic options for the 2019 novel coronavirus (2019-nCoV). Nat Rev Drug Discov. NLM (Medline); 2020;19:149–50.
2. Hung IF-N, Lung K-C, Tso EY-K, Liu R, Chung TW-H, Chu M-Y, et al. Triple combination of interferon beta-1b, lopinavir–ritonavir, and ribavirin in the treatment of patients admitted to hospital with COVID-19: an open-label, randomised, phase 2 trial. Lancet. Elsevier Ltd; 2020;395:1695–704.
3. Davoudi-Monfared E, Rahmani H, Khalili H, Hajiabdolbaghi M, Salehi M, Abbasian L, et al. Efficacy and safety of interferon β-1a in treatment of severe COVID-19: A randomized clinical trial. Antimicrob Agents Chemother. American Society for Microbiology Journals; 2020;
4. Antonelli G, Scagnolari C, Moschella F, Proietti E. Twenty-five years of type I interferon-based treatment: a critical analysis of its therapeutic use. Cytokine Growth Factor Rev. 2015;26:121–31.
5. Aricò E, Castiello L, Capone I, Gabriele L, Belardelli F. Type i interferons and cancer: An evolving story demanding novel clinical applications. Cancers (Basel). MDPI AG; 2019;11.
6. Rizza P, Moretti F, Capone I, Belardelli F. Role of type I interferon in inducing a protective immune response: perspectives for clinical applications. Cytokine Growth Factor Rev. Elsevier Ltd; 2015;26:195–201.
7. Muller U, Steinhoff U, Reis L, Hemmi S, Pavlovic J, Zinkernagel R, et al. Functional role of type

I and type II interferons in antiviral defense. Science (80-). American Association for the Advancement of Science; 1994;264:1918–21.
8. García-Sastre A. Ten Strategies of Interferon Evasion by Viruses. Cell Host Microbe. Cell Press; 2017. page 176–84.
9. Park A, Iwasaki A. Type I and Type III Interferons – Induction, Signaling, Evasion, and Application to Combat COVID-19. Cell Host Microbe. Cell Press; 2020. page 870–8.
10. Le Bon A, Schiavoni G, D'Agostino G, Gresser I, Belardelli F, Tough DF. Type i interferons potently enhance humoral immunity and can promote isotype switching by stimulating dendritic cells in vivo. Immunity. 2001;14:461–70.
11. Santini SM, Lapenta C, Logozzi M, Parlato S, Spada M, Di Pucchio T, et al. Type I interferon as a powerful adjuvant for monocyte-derived dendritic cell development and activity in vitro and in Hu-PBL-SCID mice. J Exp Med. 2000;191:1777–88.
12. Lapenta C, Santini SM, Logozzi M, Spada M, Andreotti M, Di Pucchio T, et al. Potent Immune Response against HIV-1 and Protection from Virus Challenge in hu-PBL-SCID Mice Immunized with Inactivated Virus-pulsed Dendritic Cells Generated in the Presence of IFN-α. J Exp Med. 2003;198:361–7.
13. Proietti E, Bracci L, Puzelli S, Di Pucchio T, Sestili P, De Vincenzi E, et al. Type I IFN as a natural adjuvant for a protective immune response: lessons from the influenza vaccine model. J Immunol. The American Association of Immunologists; 2002;169:375–83.
14. Aricò E, Monque DM, D'Agostino G, Moschella F, Venditti M, Kalinke U, et al. MHV-68 producing mIFNα1 is severely attenuated in vivo and effectively protects mice against challenge with wt MHV-68. Vaccine. 2011;29:3935–44.
15. Miquilena-Colina ME, Lozano-Rodríguez T, García-Pozo L, Sáez A, Rizza P, Capone I, et al. Recombinant interferon-alpha2b improves immune response to hepatitis B vaccination in haemodialysis patients: results of a randomised clinical trial. Vaccine. 2009;27:5654–60.
16. Abb J, Abb H, Deinhardt F. Age-related decline of human interferon alpha and interferon gamma production. Blut. Springer-Verlag; 1984;48:285–9.
17. Smits SL, de Lang A, Van Den Brand JMAA, Leijten LM, van IJcken WF, Eijkemans MJCC, et al. Exacerbated innate host response to SARS-CoV in aged non-human primates. Baric RS, editor. PLoS Pathog. Public Library of Science; 2010;6:e1000756.
18. Falzarano D, De Wit E, Rasmussen AL, Feldmann F, Okumura A, Scott DP, et al. Treatment with interferon-α2b and ribavirin improves outcome in MERS-CoV-infected rhesus macaques. Nat Med. 2013;19:1313–7.
19. Gruber C. Impaired interferon signature in severe COVID-19. Nat Rev Immunol. Nature Publishing Group; 2020;1–1.
20. Acharya D, Liu G, Gack MU. Dysregulation of type I interferon responses in COVID-19. Nat Rev Immunol. Springer Science and Business Media LLC; 2020;1–2.
21. van der Made CI, Simons A, Schuurs-Hoeijmakers J, van den Heuvel G, Mantere T, Kersten S, et al. Presence of Genetic Variants Among Young Men With Severe COVID-19. JAMA. American Medical Association (AMA); 2020;324:663–73.
22. Hadjadj J, Yatim N, Barnabei L, Corneau A, Boussier J, Smith N, et al. Impaired type I interferon activity and inflammatory responses in severe COVID-19 patients. Science. American Association for the Advancement of Science; 2020;369:718–24.
23. Channappanavar R, Fehr AR, Vijay R, Mack M, Zhao J, Meyerholz DK, et al. Dysregulated Type I Interferon and Inflammatory Monocyte-Macrophage Responses Cause Lethal Pneumonia in SARS-CoV-Infected Mice. Cell Host Microbe. Cell Press; 2016;19:181–93.

24. Lee JS, Shin E-C. The type I interferon response in COVID-19: implications for treatment. Nat Rev Immunol. Nature Publishing Group; 2020;1–2.
25. International Clinical Trials Registry Platform (ICTRP) [Internet]. 2020. Available from: https://www.who.int/ictrp/data/en/
26. Wang N, Zhan Y, Zhu L, Hou Z, Liu F, Song P, et al. Retrospective Multicenter Cohort Study Shows Early Interferon Therapy Is Associated with Favorable Clinical Responses in COVID-19 Patients. Cell Host Microbe. Cell Press; 2020;
27. Vilcek J. Fifty Years of Interferon Research: Aiming at a Moving Target. Immunity. 2006;25:343–8.
28. Lin F-C, Young HA. Interferons: Success in anti-viral immunotherapy. Cytokine Growth Factor Rev. Elsevier Ltd; 2014;25:369–76.
29. Nelemans T, Kikkert M. Viral innate immune evasion and the pathogenesis of emerging RNA virus infections. Viruses. MDPI AG; 2019.
30. Roth-Cross JK, Martínez-Sobrido L, Scott EP, García-Sastre A, Weiss SR. Inhibition of the alpha/beta interferon response by mouse hepatitis virus at multiple levels. J Virol. 2007;81:7189–99.
31. Channappanavar R, Fehr AR, Zheng J, Wohlford-Lenane C, Abrahante JE, Mack M, et al. IFN-I response timing relative to virus replication determines MERS coronavirus infection outcomes. J Clin Invest. American Society for Clinical Investigation; 2019;129:3625–39.
32. Yu S-Y. Gene expression profiles in peripheral blood mononuclear cells of SARS patients. World J Gastroenterol. WJG Press; 2005;11:5037.
33. Reghunathan R, Jayapal M, Hsu LY, Chng HH, Tai D, Leung BP, et al. Expression profile of immune response genes in patients with severe acute respiratory syndrome. BMC Immunol. 2005;6:2.
34. Faure E, Poissy J, Goffard A, Fournier C, Kipnis E, Titecat M, et al. Distinct immune response in two MERS-CoV-infected patients: Can we go from bench to bedside? PLoS One. Public Library of Science; 2014;9:e88716.
35. Hadjadj J, Yatim N, Barnabei L, Corneau A, Boussier J, Pere H, et al. Impaired type I interferon activity and exacerbated inflammatory responses in severe Covid-19 patients. medRxiv. Cold Spring Harbor Laboratory Press; 2020;2020.04.19.20068015.
36. Elisia I, Lam V, Hofs E, Li MY, Hay M, Cho B, et al. Effect of age on chronic inflammation and responsiveness to bacterial and viral challenges. PLoS One. Public Library of Science; 2017;12.
37. Metcalf TU, Cubas RA, Ghneim K, Cartwright MJ, Grevenynghe J Van, Richner JM, et al. Global analyses revealed age-related alterations in innate immune responses after stimulation of pathogen recognition receptors. Aging Cell. Blackwell Publishing Ltd; 2015;14:421–32.
38. Zhou F, Yu T, Du R, Fan G, Liu Y, Liu Z, et al. Clinical course and risk factors for mortality of adult inpatients with COVID-19 in Wuhan, China: a retrospective cohort study. Lancet. Elsevier Ltd; 2020;395:1054–62.
39. Sallard E, Lescure F-X, YAZDANPANAH Y, Mentre F, PEIFFER-SMADJA N, ADER F, et al. Type 1 interferons as a potential treatment against COVID-19. Antiviral Res. Elsevier; 2020;178:104791.
40. Conti P, Younes A. Coronavirus COV-19/SARS-CoV-2 affects women less than men: clinical response to viral infection. J Biol Regul Homeost Agents. 2020;34.
41. Pillai PS, Molony RD, Martinod K, Dong H, Pang IK, Tal MC, et al. Mx1 reveals innate pathways to antiviral resistance and lethal influenza disease. Science (80-). American Association for the Advancement of Science; 2016;352:463–6.

42. Liu J, Li S, Liu J, Liang B, Wang X, Wang H, et al. Longitudinal characteristics of lymphocyte responses and cytokine profiles in the peripheral blood of SARS-CoV-2 infected patients. EBioMedicine. Elsevier B.V.; 2020;55:102763.
43. Qin C, Zhou L, Hu Z, Zhang S, Yang S, Tao Y, et al. Dysregulation of immune response in patients with COVID-19 in Wuhan, China. Clin Infect Dis. Oxford University Press (OUP); 2020;
44. De Biasi S, Emilia R, Campi V, Meschiari M, Gibellini L. Marked T cell activation , senescence , exhaustion and skewing towards TH17 in patients with Covid-19 pneumonia. Res Sq. 2020;1–32.
45. Nile SH, Nile A, Qiu J, Li L, Jia X, Kai G. COVID-19: Pathogenesis, cytokine storm and therapeutic potential of interferons. Cytokine Growth Factor Rev. 2020;53:66–70.
46. Ye Q, Wang B, Mao J. The pathogenesis and treatment of the 'Cytokine Storm'' in COVID-19.' J. Infect. W.B. Saunders Ltd; 2020. page 607–13.
47. Bonafè M, Prattichizzo F, Giuliani A, Storci G, Sabbatinelli J, Olivieri F. Inflamm-Aging: Why Older Men Are the Most Susceptible to SARS-Cov-2 Complicated Outcomes. 2020;1–17.
48. Herold T, Jurinovic V, Arnreich C, Lipworth BJ, Hellmuth JC, von Bergwelt-Baildon M, et al. Elevated levels of IL-6 and CRP predict the need for mechanical ventilation in COVID-19. J Allergy Clin Immunol. Mosby Inc.; 2020;146:128-136.e4.
49. Tong M, Jiang Y, Xia D, Xiong Y, Zheng Q, Chen F, et al. Elevated Expression of Serum Endothelial Cell Adhesion Molecules in COVID-19 Patients. J Infect Dis. 2020;222.
50. Di Pucchio T, Pilla L, Capone I, Ferrantini M, Montefiore E, Urbani F, et al. Immunization of stage IV melanoma patients with Melan-A/MART-1 and gplOO peptides plus IFN-α results in the activation of specific $CD8^+$ T cells and monocyte/dendritic cell precursors. Cancer Res. 2006;66.
51. Aricò E, Castiello L, Urbani F, Rizza P, Panelli MC, Wang E, et al. Concomitant detection of IFNα signature and activated monocyte/dendritic cell precursors in the peripheral blood of IFNα-treated subjects at early times after repeated local cytokine treatments. J Transl Med. BioMed Central; 2011;9:67.
52. Urbani F, Ferraresi V, Capone I, Macchia I, Palermo B, Nuzzo C, et al. Clinical and Immunological Outcomes in High-Risk Resected Melanoma Patients Receiving Peptide-Based Vaccination and Interferon Alpha, With or Without Dacarbazine Preconditioning: A Phase II Study. Front Oncol. 2020;10:202.
53. Aricò E, Bracci L, Castiello L, Gessani S, Belardelli F. Are we fully exploiting type I Interferons in today's fight against COVID-19 pandemic? Cytokine Growth Factor Rev. Elsevier; 2020;
54. Rozera C, Cappellini GA, D'Agostino G, Santodonato L, Castiello L, Urbani F, et al. Intratumoral injection of IFN-alpha dendritic cells after dacarbazine activates anti-tumor immunity: results from a phase I trial in advanced melanoma. J Transl Med. 2015;13:139.
55. Macchia I, Urbani F, Proietti E. Immune monitoring in cancer vaccine clinical trials: Critical issues of functional flow cytometry-based assays. Biomed Res Int. 2013;2013.
56. Macchia I, La Sorsa V, Ruspantini I, Sanchez M, Tirelli V, Carollo M, et al. Multicentre Harmonisation of a Six-Colour Flow Cytometry Panel for Naïve/Memory T Cell Immunomonitoring. J Immunol Res. 2020;2020:1–15.
57. Fine JP, Gray RJ. A proportional hazards model for the subdistribution of a competing risk. J Am Stat Assoc 1999;94:496–509.
58. Antonio Ancidoni, Ilaria Bacigalupo, Guido Bellomo, Marco Canevelli, Patrizia Carbonari, Maria Grazia Carella, Annamaria Confaloni, Alessio Crestini, Fortunato (Paolo) D'Ancona, Carla Faralli, Simone Fiaccavento, Silvia Francisci, Flavia Lombardo, Eleonor NV. Survey

nazionale sul contagio COVID-19 nelle strutture residenziali e sociosanitarie REPORT FINALE.

List of Appendices

APPENDIX 1: Flow Chart of the Study

APPENDIX 2: Timeline scheme

APPENDIX 3: GANTT chart

APPENDIX 4: eCRF design

APPENDIX 5: Patient Diary and clinical record template

APPENDIX 6: Standard operating procedure for drug management

APPENDIX 1: Flow Chart of the Study

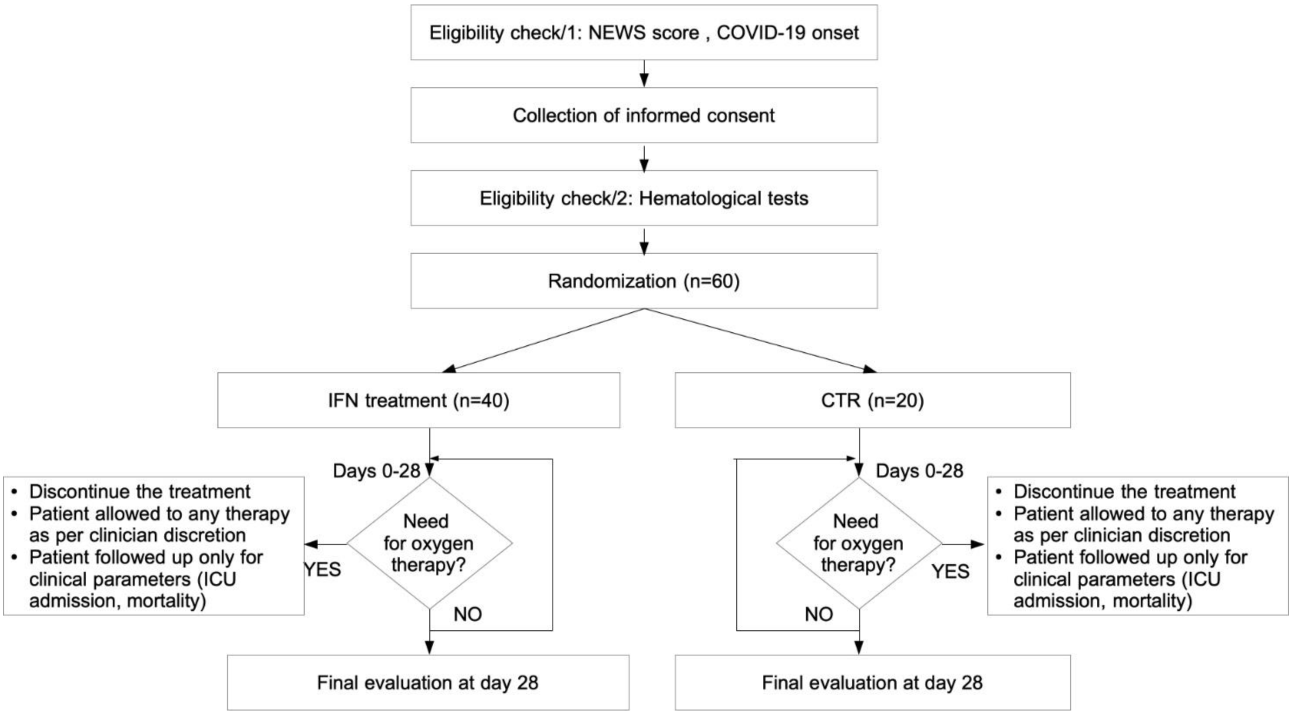

APPENDIX 2: Timeline scheme

TIMELINE SCHEME																													
**Days**	PreTx	T1	T2	T3	T4	T5	T6	T7	T8	T9	T10	T11	T12	T13	T14	T15	T16	T17	T18	T19	T20	T21	T22	T23	T24	T25	T26	T27	T28
	**Screening**	**Treatment**										**Follow-up**																	
**IFN (ARM2 only)**		x		x				x			x																		
**Procedures (both ARMS)**																													
**RT-PCR SARS-CoV 2 positivity assay**		x													x														x
**Demografic Data**	x																												
**Medical History**	x																												
**Informed Consent**	x																												
**Inclusion/Exclusion Criteria**	x																												
**Signs and symptoms**	x	x	x	x	x	x	x	x	x	x	x	x	x	x	x	x	x	x	x	x	x	x	x	x	x	x	x	x	x
**Previous/Concomitant Therapy recording**	x	x	x	x	x	x	x	x	x	x	x	x	x	x	x	x	x	x	x	x	x	x	x	x	x	x	x	x	x
**NEWS2 score assessment**	x	x	x	x	x	x	x	x	x	x	x	x	x	x	x	x	x	x	x	x	x	x	x	x	x	x	x	x	x
**Safety/Efficacy Evaluation (both ARMS)**																													
**Adverse Events recording**		x	x	x	x	x	x	x	x	x	x	x	x	x	x	x	x	x	x	x	x	x	x	x	x	x	x	x	x
**Routine laboratory test parameters**	x	x		x							x				x														x
**SARS-CoV 2 Antibodies**		x													x														x
**Exploratory labtests (ISG/CIM/SIM)**		x		x											x														

As clinically indicated, laboratory and instrumental tests can be performed on other time points that will be recorded.

APPENDIX 3: GANTT chart

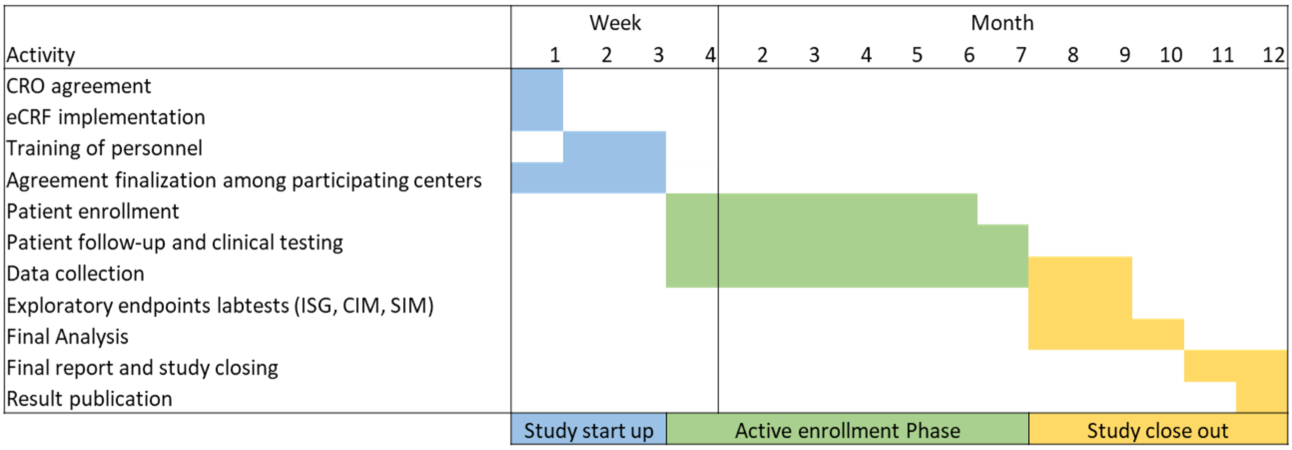

APPENDIX 4: eCRF design

The CRO will implement and validate eCRF on a Gamp 5 21 CFR part 11 compliant platform and share it among participants, endowed with different appropriate access privileges: CRO will have complete data control, while data input permission only will be assigned to local data managers.

eCRF will be designed on the basis of the following structure:

CRF sections by time of visit

Screening<br>**PreTx**	***Treatment***<br>**T1**
Id Number	Date
	Id Number
Demografic Data	IFN (ARM2 only)
Medical History	SARS-CoV-2 positivity assay
Informed Consent	Signs and symptoms
Inclusion/Exclusion Criteria	Previous/Concomitant Therapy
Signs and symptoms	NEWS2 score assessment
Previous/Concomitant Therapy	Adverse Events
NEWS2 score assessment	Routine laboratory test parameters: Procalcitonin, Reactive C protein, Protrombin time (INR), functional fibrinogen, D-dimer
Routine laboratory test parameters: Blood count, potassium, sodium, glucose, lipase, LDH, creatinine, ALT GPT, AST GOT, Bilirubin, Urea, Albumin	SARS-CoV-2 Antibodies
Randomization	Exploratory labtests (ISG/CIM/SIM)
	Study Discontinuation or Withdrawal

***Treatment* T2/T4/T5/T6/T8/T9**	***Treatment* T3**	***Treatment* T7**	***Treatment* T10**
Date<br>Id Number<br>Signs and symptoms<br>Previous/Concomitant Therapy<br>NEWS2 score assessment<br>Adverse Events<br>Study Discontinuation or Withdrawal<br>,	Date<br>Id Number<br>IFN (ARM2 only)<br>Signs and symptoms<br>Previous/Concomitant Therapy<br>NEWS2 score assessment<br>Adverse Events<br>Routine laboratory test parameters: blood count<br>Exploratory labtests (ISG/CIM/SIM)<br>Study Discontinuation or Withdrawal	Date<br>Id Number<br>IFN (ARM2 only)<br>Signs and symptoms<br>Previous/Concomitant Therapy<br>NEWS2 score assessment<br>Adverse Events<br>Study Discontinuation or Withdrawal	Date<br>Id Number<br>IFN (ARM2 only)<br>Signs and symptoms<br>Previous/Concomitant Therapy<br>NEWS2 score assessment<br>Adverse Events<br>Routine laboratory test parameters: Blood count, potassium, sodium, glucose, lipase, LDH, creatinine, ALT GPT, AST GOT, Bilirubin, Urea, Albumin, Reactive C protein, Protrombin time (INR), functional fibrinogen, D-dimer, Procalcitonin<br>Study Discontinuation or Withdrawal

Follow up **T11/T12/T13/T15/T16/T17/T18/T19/T20/T21/T22/T23/T24/T25/T26/T27**	***Follow up*** **T14**	***Follow up*** **T28**
Date<br>Id Number<br>Previous/Concomitant Therapy<br>NEWS2 score assessment<br>Adverse Events<br>Study Discontinuation or Withdrawal	Date<br>Id Number<br>SARS-CoV-2 positivity assay<br>Signs and symptoms<br>Previous/Concomitant Therapy<br>NEWS2 score assessment<br>Adverse Events<br>Routine laboratory test parameters: Blood count, potassium, sodium, glucose, lipase, LDH, creatinine, ALT GPT, AST GOT, Bilirubin, Urea, Albumin,<br>SARS-CoV-2 Antibodies<br>Exploratory labtests (~~ISG~~/CIM/SIM)<br>Study Discontinuation or Withdrawal	Date<br>Id Number<br>SARS-CoV-2 positivity assay<br>Signs and symptoms<br>Previous/Concomitant Therapy<br>NEWS2 score assessment<br>Adverse Events<br>Routine laboratory test parameters: Blood count, potassium, sodium, glucose, lipase, LDH, creatinine, ALT GPT, AST GOT, Bilirubin, Urea, Albumin, Reactive C protein, Protrombin time (INR), functional fibrinogen, D-dimer, Procalcitonin<br>SARS-CoV-2 Antibodies<br>Study Discontinuation or Withdrawal

CRF - section data		
**SARS-CoV-2 positivity assay**	date	RT-PCR: Gene name, CT number, Laboratory (INMI or other, to be specified); rapid antigen test: positive/negative (executed by: operator ID)
**Demografic Data**	date	Sex at birth, Date of birth, Race/Ethnicity; i) hospitalized, ii) RSA, iii) home patient
**Medical History**	date	see TABLE 1
**Informed Consent**	signature date	Y/N
**Inclusion/Exclusion Criteria**	date	see TABLE 2
**Previous/Concomitant Therapy**	date	Any drug/medicament name, reason for use, dose, frequency, duration of consumption
**NEWS2 score assessment**	date	SCORE, Systolic and diastolic arterial pressure, heart rate (HR), respiratory rate (RR), systemic body temperature, ACVPU, SpO_2
**Randomization**	date	ARM1/ARM2, random number
**Signs and symptoms**		see TABLE 3
**IFN (ARM2 only)**		Y/N, expiration date, batch number
**Adverse Events**		TABLE 4 AE and any other AE will be recorded using NCI Common Terminology Criteria for adverse Events (CTCAE) version 5. SUSAR will be recorded in specific CRF.
**Routine laboratory test parameters**		see TABLE 5
**SARS-CoV-2 Antibodies**		anti-S IgG, anti-N IgG, anti-S IgM, anti S IgA, neutralizing anti-S Ab titer
**Exploratory labtests (ISG/CIM/SIM)**		ISG: IFI44L, IFI27, RSAD2, SIGLEC1, IFIT1, IS15. CIM: see TABLE 6. SIM: see TABLE 7.
**Study Discontinuation or Withdrawal**		Y/N, reason: withdrawal of subjects for non-compliance/adherence, for AE, consent withdrawal, other (to be specified)

**TABLE 1 - Medical History**		
		Tobacco, Alcohol, other recreational drug use (dose, frequency, duration of comsumption)<br>Flu vaccine (in the last year): Y/N, date
By System	Respiratory	Chronic pulmonary disease, Asthma, Tuberculosis (active/previous), other (to be specified)
	Cardiovascular/Circulatory	Chronic cardiac disease (not hypertension), Hypertension, other (to be specified)
	Musculoskeletal	to be specified
	Endocrine	to be specified
	Hematopoietic	to be specified
	Nervous	Chronic neurological disorder, other (to be specified)
	Dermatological	to be specified
	Integumentary System/Exocrine System	to be specified
	Genitourinary	Chronic kidney disease, other (to be specified)
	Lymphatic System/Immune System	to be specified
	Digestive	Chronic liver disease, other (to be specified)
	Metabolic disease	Diabetes, other (to be specified)
	Ear, Nose, Throath	to be specified
	Psychiatric disease	to be specified
	Allergy	to be specified
	Malignant neoplasm	to be specified
	Infectious disease	HIV, HCV, other (to be specified)
	Other (to be specified)	

Note: For any disease, onset date and duration as well as indication about disease current status will be recorded.

TABLE 2 - Inclusion/Exclusion Criteria	
Inclusion criteria (all required):	
≥ 65 years of age at time of enrolment	Y/N
Subject has laboratory-confirmed SARS-CoV-2 infection as determined by PCR, in any specimen < 72 hours prior to randomization	Y/N
Subject (or legally authorized representative) provides written informed consent prior to initiation of any study procedures	Y/N
Subject understands and agrees to comply with planned study procedures	Y/N
Subject agrees to the collection of nasopharyngeal swabs and venous blood samples per protocol	Y/N
Being symptomatic for less than 7 days before starting therapy	Y/N
NEWS2 score ≤2	Y/N
Exclusion criteria:	
Hospitalized patients with illness of any duration, and at least one of the following: Clinical assessment (evidence of rales/crackles on exam) AND SpO2 ≤ 94% on room air at rest or after walking test, OR Acute respiratory failure requiring mechanical ventilation and/or supplemental oxygen.	Y/N
Patients currently using interferon-beta (e.g., multiple sclerosis patients)	Y/N
Patients with chronic kidney diseases	Y/N
Known allergy or hypersensitivity to interferon (including asthma)	Y/N
Any autoimmune disease (based on the anamnesis)	Y/N
Patients with signs of dementia or neurocognitive disorders	Y/N
Patients with current severe depression and/or suicidal ideations	Y/N
Being concurrently involved in another trial for COVID-19	Y/N
HIV infection (based on the anamnesis)	Y/N
Use of any antiretroviral medication	Y/N
Impaired renal function (eGFR calculated by CKD-EPI Creatinine equation < 30 ml/min)	Y/N
Presence of other severe diseases impairing life expectancy (e.g. patients are not expected to survive 28 days given their pre-existing medical condition)	Y/N
Any physical or psychological impediment in a patient that could let the investigator to suspect his/her poor compliance	Y/N
Lack or withdrawal of informed consent	Y/N

TABLE 3 - Signs and symptoms
History of fever
Lower chest indrawing
Cough (with sputum production/with emoptysis)
Headache
Altered consciousness/confusion
Seizures
Sore throat
Abdominal pain
Runny nose
Vomiting/nausea
Wheezing
Diarrhoea
Chest pain
Conjunctivitis
Muscle aches
Skin rash
Joint pain (arthralgia)
Skin ulcers
Fatigue/malaise
Lymphadenopathy
Loss of taste
Inability to walk
Loss of smell
Bleeding (ischaemic stroke, entracerebral haemorrhage)
Shortness of breath

Note: If present, onset date and duration as well as indication about sign or symptom current status will be recorded.

TABLE 4 - REBIF common AE	
Very common (≥1/10)	Common (≥1/100 to <1/10)
Neutropenia, lymphopenia, leukopenia, thrombocytopenia, anaemia	Diarrhoea, vomiting, nausea
Asymptomatic transaminase increase	Severe elevations in transaminases
	Pruritus, rash, erythematous rash, maculo-papular rash, alopecia
Headache	
Injection site inflammation, injection site reaction, influenza-like symptoms	
	Myalgia, arthralgia
	Depression, insomnia
	Injection site pain, fatigue, rigors, fever

TABLE 5 - Routine laboratory test parameters	
Haemoglobin	
Haematocrit	
Full Blood count	ALT/SGPT
Creatinine	
Sodium	AST/SGOT
Potassium	ESR
Procalcitonin	Total bilirubin
	Urea (BUN)
Glucose	Albumin
Lipase	
LDH	
PT (seconds)	INR
fibrinogen	
D-dimer	CRP

TABLE 6 - Cellular Immune Monitoring		
leu_linfociti	CD4_CM	Vd2_TD
lym_CD3	CD4_EM	Vd2_EM
CD3_CD4SP	CD4_N	Vd2_N
CD3_CD8SP	CD4_TD	Vd2_CM
CD8_CM	CD4_CD28pos27neg	lym_CD19
CD8_EM	CD4_CD28pos27pos	lym_NK
CD8_N	CD4_CD28neg27neg	lym_NKT
CD8_TD	CD4_CD28neg27pos	leu_CD14pos
CD8_CD28pos27neg	CD4_CD57pos27neg	leu_Treg
CD8_CD28pos27pos	CD4_CD57posPD1neg	lym_Treg
CD8_CD28neg27neg	CD4_CD57posPD1pos	CD3_Treg
CD8_CD28neg27pos	CD4_CD57negPD1pos	CD4_Treg
CD8_CD57pos CD27neg	gpSpike_CD8	Treg_CD45RAnegCD39neg
CD8_CD57posPD1neg	Vd2	Treg_CD45RAnegCD39pos
CD8_CD57posPD1pos		Treg_CD45RAposCD39neg
CD8_CD57negPD1pos		Treg_CD45RAposCD39pos

TABLE 7 - Systemic Inflammation Markers	
IL-2	IP-10
IL-7	MCP1
IL-10	MIP1a
G-CSF	VCAM-1
ICAM-1	VAP-1
Fractalkine	TNF-α

APPENDIX 5: Patient Diary

DIARIO CLINICO

NOME: ____________ COGNOME: _____________ DATA DI NASCITA:__________

CODICE IDENTIFICATIVO: ____________________

PER COMUNICAZIONI URGENTI CHIAMARE:_____________________________

STUDIO CLINICO: “**Valutazione dell’attività antivirale e immunomodulatoria di Interferone-Beta in pazienti COVID-19 anziani**”

Numero EUDRACT: 2020-003872-42

Promotore: Istituto di Farmacologia Traslazionale – Consiglio Nazionale delle Ricerche

Sperimentatore Principale: Dott Emanuele Nicastri

Telefono: xxxxxxxxxxxxx

**GIORNO**													
**ORARIO**													
**TEMPERATURA CORPOREA**													
**SATURAZIONE**													
**FREQUENZA CARDIACA**													
**PRESSIONE**													
**FREQUENZA RESPIRATORIA**													
**NECESSITA OSSIGENO?**													
**È VIGILE?**													

APPENDIX 6: Standard operating procedure for drug management

**Istituto di Farmacologia Traslazionale -CNR**		ANTIICIPATE-SOP-01<br>rev. 00 pag. 1 di 6
**MODALITÀ DI APPROVVIGGIONAMENTO E SOMMINISTRAZIONE DEL FARMACO REBIF TRAMITE INIETTORE REBISMART**		

Documento Redatto da: Luciano Castiello Firma e data ___________________

Documento Approvato da:

Ruolo	Nominativo	Firma e Data
Sperimentatore Principale	Dr. Emanuele Nicastri	
Coordinatore Scientifico	Dr. Filippo Belardelli	
Project Manager - FullCRO	Dr.ssa Moira Cordisco	
ISS	Dr.ssa Eleonora Aricò	
Farmacia INMI "Lazzaro Spallanani	Dr.ssa Silvia Murachelli	

Lista di distribuzione:

Ruolo	Nominativo	N° copie	Firma e Data
Sperimentatore Principale	Dr. Emanuele Nicastri	1	
Coordinatore Scientifico	Dr. Filippo Belardelli	1	
Project Manager - FullCRO	Dr.ssa Moira Cordisco	1	
ISS	Dr.ssa Eleonora Aricò	1	
Farmacia INMI "Lazzaro Spallanani	Dr.ssa Silvia Murachelli	1	

1. Scopo

La presente procedura descrive le operazioni che gli sperimentatori devono eseguire per recuperare il farmaco e per utilizzare correttamente l'autoiniettore Rebismart nella sperimentazione clinica ANTIICIPATE sia nel setting ospedaliero che in quello non-ospedaliero.

2. Abbreviazioni e definizioni

Farmaco: Rebif (Interferon beta-1a)

Farmacia: Farmacia dell'Istituto Nazionale per le Malattie Infettive "Lazzaro Spallanzani"

CRO: FullCRO

Promotore: Istituto di Farmacologia Traslazionale

3. Approvviggionamento del farmaco e dell'iniettore

Per i soli pazienti rientranti nel braccio di trattamento, il giorno previsto per l'inizio del trattamento l'investigatore o un suo delegato deve presentarsi presso la Farmacia e richiedere una dose di farmaco e un dispositivo per l'autoiniezione del farmaco utilizzando il modulo Allegato 1. Il modulo originale deve essere conservato dalla Farmacia fino al ritorno del dispositivo per l'autoiniezione, dopodichè il modulo viene conservato nella cartella clinica del paziente.

Per i soli pazienti non ospedalizzati, l'investigatore dovrà accertarsi di essere inoltre in possesso di:

- Sei aghi Serofine 29G, 30G o 31G;
- Un contenitore per rifiuti sanitari pericolosi a rischio infettivo (taglienti e pungenti);
- Un diario clinico del paziente (fornito dal Promotore);
- Uno sfingomanometro digitale, un termometro e un saturimetro per la misurazione dei parametri vitali (fornito dal Promotore);
- Un manuale d'uso del Rebismart (da usare in caso di necessità);
- Salviettine o tamponcini imbevuti di alcol o batuffoli di ovatta e alcol per frizione;
- Cerotti (classici e a rocchetto)

4. Attivazione e settaggio dell'iniettore Rebismart e caricamento cartuccia Rebif

4.1 Inserimento pile

In caso il dispositivo abbia le batterie scariche, prelevare il dispositivo Rebismart dalla scatola contenitore e indossare il cinturino per impedire che il dispositivo possa cadere accidentalmente. Svitare la vite del coperchio dell'alloggiamento pile con un cacciavite e far scivolare verticalmente il coperchio. Inserire 4 pile al litio nuove. Verificare che siano orientate come mostrato sul dispositivo e stringere la vite del coperchio dell'alloggiamento delle pile per chiuderlo. Una sequenza illustrata della attività da eseguire è riportata nell'allegato 2.

4.2 Settaggio Rebismart

4.2.1 Settaggio cartuccia e dose

Prelevare il dispositivo Rebismart dalla scatola contenitore e indossare il cinturino per impedire che il dispositivo possa cadere accidentalmente. Accendere RebiSmart e premere il pulsante 'Menu'. Scorrere su 'Impostaz. iniezione' e premere il pulsante 'Apri' per selezionare. Sarà visualizzata una schermata di avvertenza e selezionare 'Si'.
Selezionare 'Cartuccia' premendo su 'Cambia' e scorrere al dosaggio della cartuccia 22mcg. Premere 'OK' per selezionare e confermare la selezione.

Dal menù 'Impostaz. Iniezione' scorrere e selezionare 'Riduzione dose' e premere il pulsante 'Cambia' per selezionare. Inserire il codice PIN del dispositivo. Il dispositivo visualizzerà il menu 'Riduzione dose'. Selezionare '50% della dose'. Premere 'Ok' e confermare la selezione. Premere due volte il pulsante 'Esci' per tornare alla schermata informativa.

4.2.2 Settaggio ago

Prelevare il dispositivo Rebismart dalla scatola contenitore e indossare il cinturino per impedire che il dispositivo possa cadere accidentalmente. Accendere RebiSmart. E premere il pulsante 'Menu'. Scorrere su 'Impostaz. pers.' e premere il pulsante 'Apri' per selezionare. Si apre la schermata di avvertenza e selezionare 'Si'. Selezionare 'Velocità ago' premendo su 'Cambia'. Selezionare velocità 'Media'. Premere 'OK' per selezionare.

Scorrere su 'Velocità iniez.' e premere il pulsante 'Cambia'. Selezionare velocità 'Media'. Premere 'OK' per selezionare. Scorrere in basso su 'Profondità iniez.' e premere il pulsante 'Cambia'. Selezionare '4 mm' e premere 'OK' per selezionare.

Scorrere in basso su 'Durata iniezione' e premere il pulsante 'Cambia'. Selezionare '3 secondi' premere 'OK' per selezionare. Premere due volte il pulsante 'Esci/Esci' per tornare alla schermata informativa.

Premere il pulsante 'Menu', scorrere su 'Impostaz. iniezione' e premere il pulsante 'Apri' per selezionare. Scorrere su 'Tipo di ago' e premere il pulsante 'Cambia' per selezionare. Selezionare il tipo di ago utilizzato. Premere 'OK' per selezionare. Premere due volte il pulsante 'Esci' per tornare alla schermata informativa.

4.3 Caricamento cartuccia

Prelevare il dispositivo Rebismart dalla scatola contenitore e indossare il cinturino per impedire che il dispositivo possa cadere accidentalmente. Estrarre la cartuccia dal confezionamento secondario. Accendere RebiSmart tenendo premuto il pulsante 'Acceso' fino a che compare la schermata di "Benvenuto (Ciao)". Premere su 'Inizio' e aprire lo sportello dell'alloggiamento della cartuccia facendo scorrere verso l'alto il pulsante posto sul lato sinistro del dispositivo. Inserire la cartuccia di Rebif nell'alloggiamento cartuccia con la parte metallica rivolta verso il basso. Chiudere lo sportello dell'alloggiamento cartuccia fino ad udire un "clic". Una sequenza illustrata della attività da eseguire è riportata nell'allegato 3. Staccare dal confezionamento secondario la parte staccabile dell'etichetta e applicarla sul Rebismart..

5. Somministrazione farmaco

5.1 Preparazione somministrazione

Prima di iniziare, estrarre RebiSmart. dal frigorifero e dalla scatola di conservazione almeno 30 minuti prima dell'utilizzo previsto. Disporre, su una superficie stabile, come per esempio un tavolo, quanto segue:

• RebiSmart. contenente una cartuccia di Rebif in posizione verticale;
• Ago Serofine™ (29G, 30G o 31G, in base alla prescrizione);
• Salviettine o tamponcini imbevuti di alcol o batuffoli di ovatta e alcol per frizione;
• Piccolo cerotto;
• Contenitore per rifiuti sanitari pericolosi a rischio infettivo (taglienti e pungenti);

Sanitizzare i guanti accuratamente. Indossare il cinturino per impedire che il dispositivo possa cadere accidentalmente. Accendere RebiSmart tenendo premuto il pulsante 'Acceso' fino a che compare la schermata di "Benvenuto (Ciao)". Premere il pulsante 'Inizio'.

Qualora RebiSmart visualizzi il messaggio "Meno di 48 ore dall'ultima iniezione. Procedere con l'iniezione", selezionare 'Si'. Prelevare un ago e rimuovere il sigillo di sterilità.

Inserire il cappuccio che contiene l'ago direttamente nell'alloggiamento dell'ago fino a che si blocca con un 'clic'. Togliere il cappuccio dell'ago spingendolo di lato fino a che non venga rimosso e conservare il cappuccio. Una sequenza illustrata della attività da eseguire è riportata nell'allegato 4.

5.2 Somministrazione farmaco

Posizionare RebiSmart sulla cute in posizione verticale nel sito di iniezione più idoneo (in base alle caratteristiche del paziente scegliere tra la parte esterna superiore delle braccia, la zona periombelicale dell'addome, la parte anteriore delle cosce). Assicurarsi che il sensore cutaneo sia completamente a contatto con la cute. Quando RebiSmart è posizionato correttamente sulla cute, la luce del pulsante di iniezione diventa verde e RebiSmart emette un bip.

Premere il pulsante per iniziare l'iniezione. La spia del pulsante di iniezione verde durante l'iniezione lampeggia. Tenere RebiSmart a contatto con la pelle per tutta la durata dell'iniezione.

Al termine dell'iniezione, la spia del pulsante verde si spegne e RebiSmart emette due bip.

Sollevare delicatamente RebiSmart dalla cute. Premere su 'OK' per confermare che l'iniezione è stata praticata correttamente.
Registrare sul diario clinico del paziente le informazioni sulla somministrazione.

5.3 Eliminazione ago e spegnimento Rebismart

Inserire il cappuccio vuoto direttamente nell'alloggiamento dell'ago fino a che si blocca con un 'clic'. RebiSmar. emette un bip. Premere e mantenere premuto il pulsante di rilascio dell'ago fino a che RebiSmart emette due bip. Togliere il cappuccio contenente l'ago spingendolo di lato fino a che si stacca per essere facilmente rimosso.
Controllare l'interno del cappuccio dell'ago per vedere l'ago rimosso. Gettare gli aghi usati nel contenitore per rifiuti sanitari pericolosi a rischio infettivo (taglienti e pungenti).

Premere e tenere premuto il pulsante 'Spento' fino a che RebiSmart si spegne e la schermata informativa si chiude.

6. Conservazione del farmaco e dell'iniettore

Al termine dell'iniezione riposizionare RebiSmart in posizione verticale nella sua custodia all'interno del frigorifero.

7. Recupero dell'iniettore e riconsegna

Al termine della quarta iniezione, eliminare la cartuccia in uso. Prima di spegnere Rebismart, premere il pulsante 'Menu'. Scorrere in basso su 'Rimuovere cartuc.' e premere il pulsante 'Apri'. Premere su 'Si' per confermare la selezione. Attendere che Rebismart visualizza il messaggio 'Aprire sportello alloggiamento cartuccia' ed emette due bip. Far scorrere verso l'alto il pulsante dello sportello alloggiamento cartuccia e rimuovere la cartuccia. Gettare la cartuccia nel contenitore

per rifiuti sanitari pericolosi a rischio infettivo (taglienti e pungenti). Selezionare 'No' sul Rebismart e spegnerlo. Rimuovere il confezionamento secondario dalla custodia del Rebismart.

Riconsegnare il Rebismart alla Farmacia, registrando la consegna sul modulo Allegato 1 utilizzato per il ritiro.

8. Allegati

Allegato 1: Modulo richiesta ritiro/riconsegna Rebif e Rebismart

Allegato 2: Schema dei passaggi da effettuare per la sostituzione pile

Allegato 3: Schema dei passaggi da effettuare per l'inserimento di una cartuccia di Rebif

Allegato 4: Schema dei passaggi da effettuare per somministrare il farmaco

	**Istituto di Farmacologia Traslazionale -CNR**	ANTIICIPATE-SOP-01<br>rev. 00 ALLEGATO 1<br>pag. 1 di 1
**MODULO RICHIESTA RITIRO/RICONSEGNA REBIF E REBISMART**		

DATI RICHIEDENTE

NOME E COGNOME:_________________________ UNITÀ: INMI USCAR

RICHIEDE n° 1 cartuccia/e Rebif 66 mcg e n° 1 dispositivo Rebismart per effettuare il trattamento al paziente

Codice ____________ **Data di Nascita:** ______________

DATA:_______________ FIRMA: _____________________

Spazio da compilare a cura della Farmacia

Numero lotto Rebif:___________________ Data di scadenza:_________________

Numero seriale cartuccia:_______________

Identificativo Rebismart:________________

Nome e Cognome:_____________________ Data:__________ Ora:___________

Firma:_______________________________

Spazio da compilare alla riconsegna del dispositivo Rebismart a cura della Farmacia

Identificativo dispositivo Rebismart riconsegnato: ________________

Nome e Cognome:_____________________ Data:__________ Ora:___________

Firma:_______________________________

Istituto di Farmacologia Traslazionale -CNR — *ANTIICIPATE-SOP-01 v. 00 ALLEGATO 2 pag. 1 di 1*

SCHEMA DEI PASSAGGI DA EFFETTUARE PER LA SOSTITUZIONE PILE

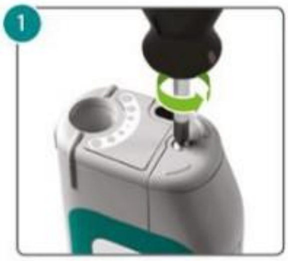

Svitare la vite del coperchio dell'alloggiamento pile con un cacciavite.

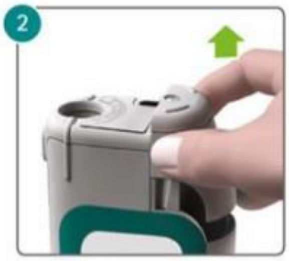

Afferrare il coperchio sui due lati e farlo scivolare via.

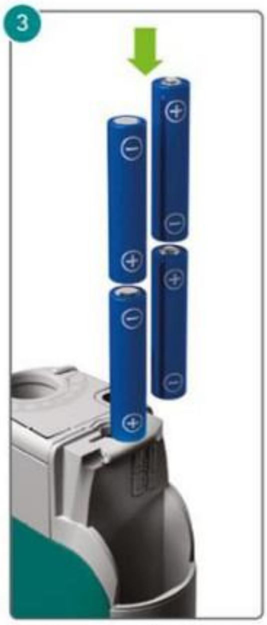

Inserire 4 pile al litio nuove. Verificare che siano orientate come mostrato sul dispositivo.

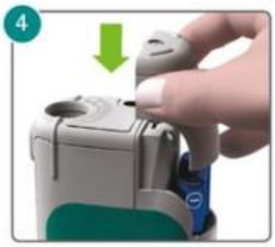

Far scorrere il coperchio dell'alloggiamento delle pile nella posizione di chiusura, verificando che entri nelle fessure.

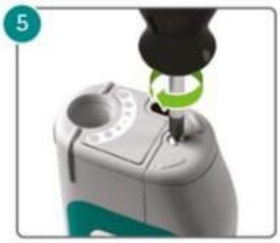

Stringere la vite del coperchio dell'alloggiamento delle pile per chiuderlo.

SCHEMA DEI PASSAGGI DA EFFETTUARE PER L'INSERIMENTO DI UNA CARTUCCIA DI REBIF

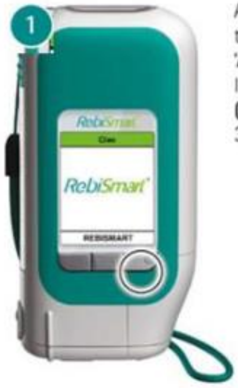

Accendere RebiSmart® tenendo premuto il pulsante '**Acceso**' fino a che compare la schermata di **"Benvenuto (Ciao)"**, in genere dopo 3-5 secondi.

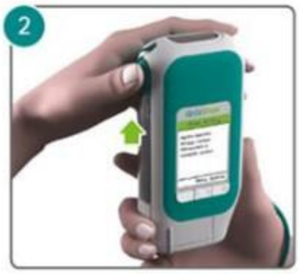

Premere su '**Inizio**' e aprire lo sportello dell'alloggiamento della cartuccia facendo scorrere il pulsante verso l'alto.

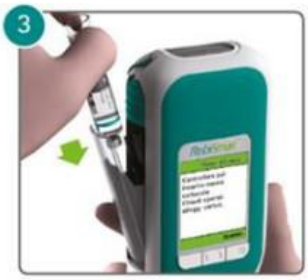

Inserire una nuova cartuccia di Rebif® nell'alloggiamento cartuccia, verificando che la parte metallica sia rivolta verso il basso.

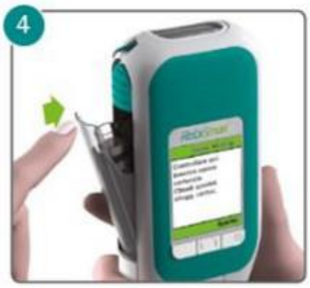

Chiudere lo sportello dell'alloggiamento cartuccia fino ad udire un "clic".

**Istituto di Farmacologia Traslazionale -CNR**	ANTIICIPATE-SOP-01<br>v. 00 ALLEGATO 4 pag. 1 di 3

SCHEMA DEI PASSAGGI DA EFFETTUARE PER SOMMINISTRARE IL FARMACO

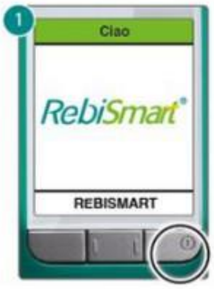

Accendere il dispositivo premendo e tenendo premuto il pulsante **'Acceso'** fino a che compare la schermata di **"Benvenuto (Ciao)"**, in genere dopo 3-5 secondi.

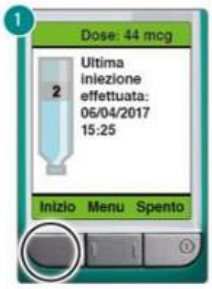

Premere il pulsante **'Inizio'**.

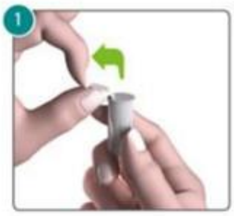

Verificare che la misura in gauge (G) indicata sulla scatola degli aghi Serofine™ corrisponda a quella indicata nella schermata di RebiSmart®.

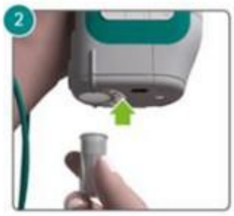

Inserire il cappuccio che contiene l'ago direttamente nell'alloggiamento dell'ago fino a che si blocca con un 'clic'.

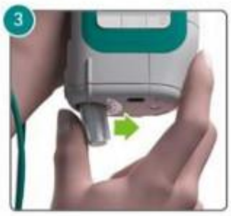

Togliere il cappuccio dell'ago spingendolo di lato fino a che venga rimosso.

SCHEMA DEI PASSAGGI DA EFFETTUARE PER SOMMINISTRARE IL FARMACO

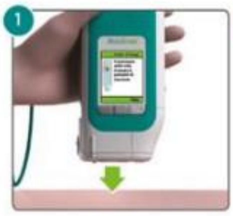

Posizionare RebiSmart® sulla cute in posizione verticale nel sito di iniezione preparato come indicato dal medico o dall'infermiere.

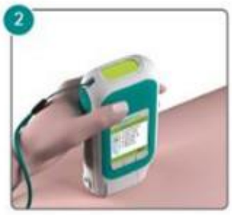

Quando RebiSmart® è posizionato correttamente sulla cute, la luce del pulsante di iniezione diventa verde e RebiSmart® emette un bip. 1

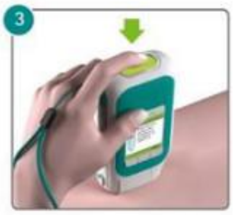

Premere il pulsante per iniziare l'iniezione.

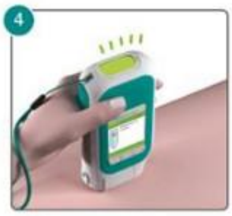

La spia del pulsante di iniezione verde durante l'iniezione lampeggia. Tenere RebiSmart® a contatto con la pelle per tutta la durata dell'iniezione. Non è necessario mantenere premuto il pulsante di iniezione.

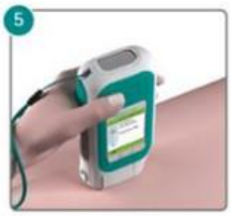

Al termine dell'iniezione, la spia del pulsante verde si spegne e RebiSmart® emette due bip. 2

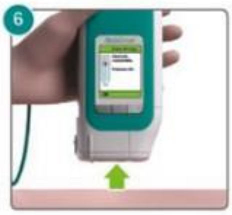

Sollevare delicatamente RebiSmart® dalla cute.

Premere su '**OK**' per confermare che l'iniezione è stata praticata correttamente.

Istituto di Farmacologia Traslazionale -CNR ANTIICIPATE-SOP-01
v. 00 ALLEGATO 4 pag. 3 di 3

SCHEMA DEI PASSAGGI DA EFFETTUARE PER SOMMINISTRARE IL FARMACO

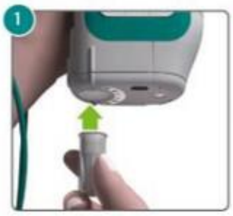

Verificare che il cappuccio dell'ago sia vuoto.

Inserire il cappuccio vuoto direttamente nell'alloggiamento dell'ago fino a che si blocca con un 'clic'. RebiSmart® emette un bip 1

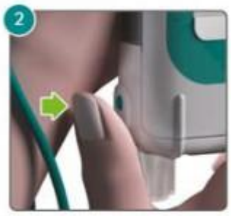

Premere e mantenere premuto il pulsante di rilascio dell'ago fino a che RebiSmart® emette due bip. 2

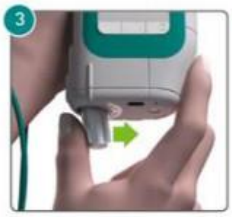

Togliere il cappuccio contenente l'ago spingendolo di lato fino a che si stacca per essere facilmente rimosso.

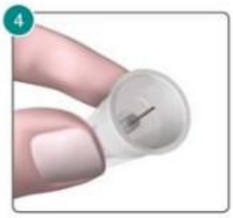

Controllare l'interno del cappuccio dell'ago per vedere l'ago rimosso come mostrato nell'immagine.
